# Ionic Liquid-Based Materials for Biomedical Applications

**DOI:** 10.3390/nano11092401

**Published:** 2021-09-15

**Authors:** Daniela Maria Correia, Liliana Correia Fernandes, Margarida Macedo Fernandes, Bruno Hermenegildo, Rafaela Marques Meira, Clarisse Ribeiro, Sylvie Ribeiro, Javier Reguera, Senentxu Lanceros-Méndez

**Affiliations:** 1 Centre of Physics, University of Minho, 4710-058 Braga, Portugal; lfernandes@fisica.uminho.pt (L.C.F.); margaridafernandes@fisica.uminho.pt (M.M.F.); rafaelammeira95@gmail.com (R.M.M.); cribeiro@fisica.uminho.pt (C.R.); sribeiro@fisica.uminho.pt (S.R.); 2 Centre of Chemistry, University of Trás-os-Montes e Alto Douro, 5000-801 Vila Real, Portugal; 3 CEB—Centre of Biological Engineering, University of Minho, 4710-057 Braga, Portugal; 4 BCMaterials, Basque Center for Materials, Applications and Nanostructures, UPV/EHU Science Park, 48940 Leioa, Spain; bruno.hermenegildo@bcmaterials.net; 5 IB-S—Institute for Research and Innovation on Bio-Sustainability, University of Minho, 4710-057 Braga, Portugal; 6 IKERBASQUE, Basque Foundation for Science, 48009 Bilbao, Spain

**Keywords:** biomedical applications, ionic liquids, IL-polymer based materials

## Abstract

Ionic liquids (ILs) have been extensively explored and implemented in different areas, ranging from sensors and actuators to the biomedical field. The increasing attention devoted to ILs centers on their unique properties and possible combination of different cations and anions, allowing the development of materials with specific functionalities and requirements for applications. Particularly for biomedical applications, ILs have been used for biomaterials preparation, improving dissolution and processability, and have been combined with natural and synthetic polymer matrixes to develop IL-polymer hybrid materials to be employed in different fields of the biomedical area. This review focus on recent advances concerning the role of ILs in the development of biomaterials and their combination with natural and synthetic polymers for different biomedical areas, including drug delivery, cancer therapy, tissue engineering, antimicrobial and antifungal agents, and biosensing.

## 1. Introduction

### 1.1. Ionic Liquids for Biomedical Applications

**Figure 1:**
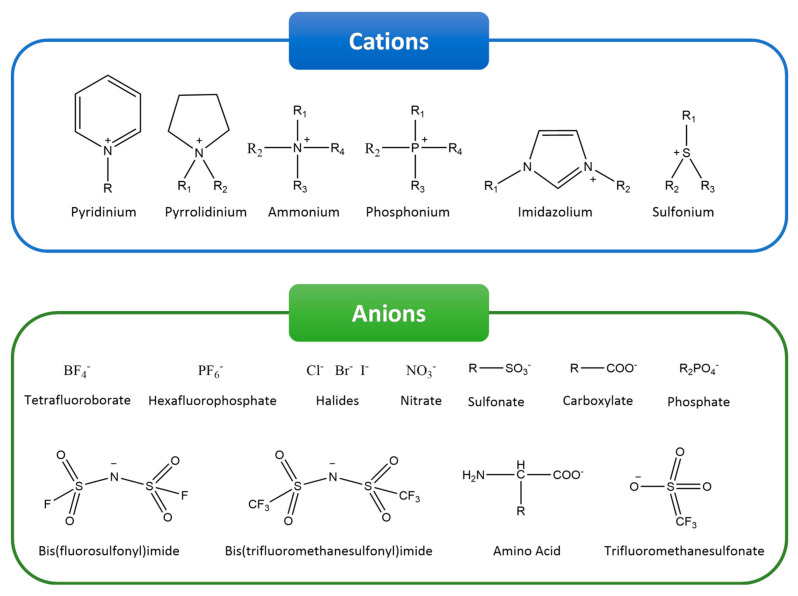
Some common cations and anions present in ionic liquids. Typical bases of the cations are ammonium, imidazolium, sulfonium, piperidinium, and pyridinium ions. Halides, tetrafluoroborate, nitrate, sulfonate, carboxylate, phosphate, and amino acid, among others, serve as anionic bases for the preparation of ILs. Adapted from [2].

Ionic liquids (ILs) have been applied in a wide range of areas such as sensors and actuators. In recent years, however, ILs have also been gaining special attention in the biomedical field, where they provide important advances in novel pharmaceutics and medical strategies. ILs are commonly defined as salts entirely composed of organic cations and organic/inorganic anions with low melting temperatures [1]. Due to the high number of possible cations and anions, a large amount of different ILs with a variety of functionalities can be synthesized (Figure 1) [1,2]. Further, their unique properties make ILs an interesting approach for the development of polymer-based hybrid materials. Particularly for biomedical applications, ILs are known for their biological activity, such as cytotoxicity against cancer cells, and antimicrobial properties, being also employed in drug delivery systems [3]. Additionally, due to their unique and interesting properties, ILs have been explored and applied in a large variety of areas, including sensors, actuators, batteries and fuel cells, separation, and catalysis, among others [1].

Some of the interesting physical-chemical properties of ILs include viscosity [4,5], melting temperature [6], solubility, stability at high temperatures, and surface activity [7], which are important for developing suitable materials for biomedical applications. It is noteworthy that deep eutectic solvents (DESs), formed by the mixture of a hydrogen bond acceptor and a donor, show similar properties to ILs and have been also used for biomedical applications. However, DESs have been replaced by ILs in the most recent studies [8,9,10].

**Figure 2:**
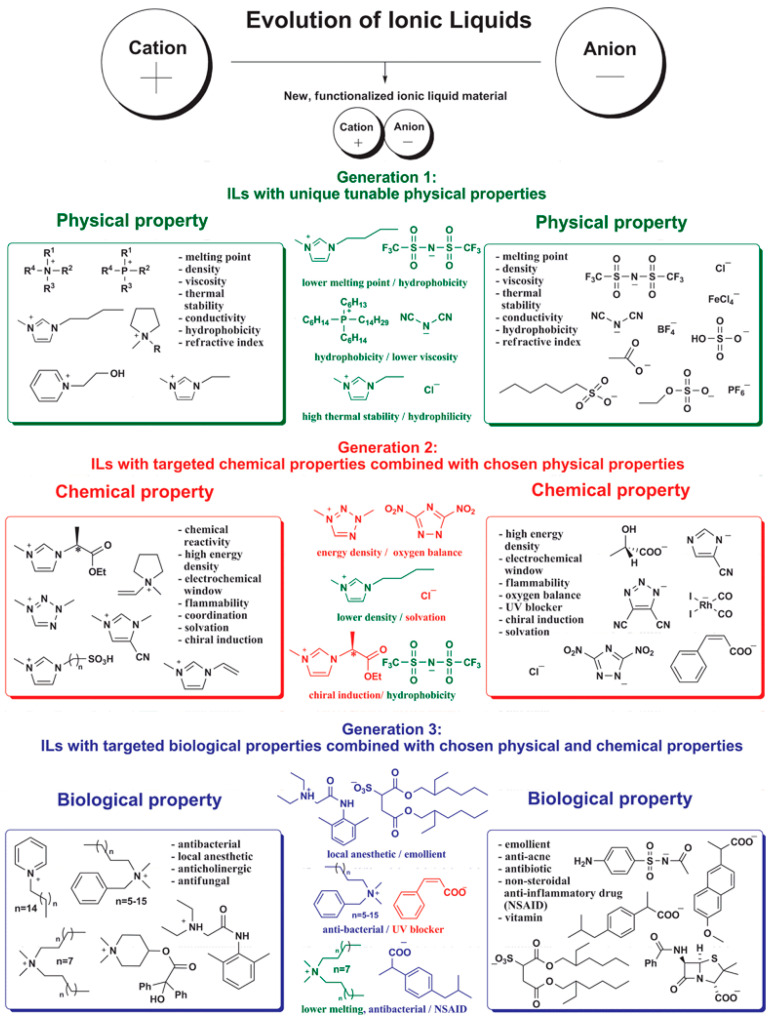
Evolution of the scientific focus on ILs from unique physical through unique chemical and now biological property sets. Reprinted with permission from [12].

Figure 2 shows representative IL properties, attending to the three generations of ILs [11,12]. The first IL was discovered by Paul Walden in 1914 [13], being the first ILs generation reported in the 1970s–1980s [1]. ILs are characterized by their low vapor pressure, low melting point, high thermal stability with availability to replace some volatile and environmentally undesirable organic agents/solvents. First generation ILs (e.g., 1-butyl-3-methylimidazolium tetrafluoroborate ([Bmim][BF_4_]) are typically poorly biodegradable and ecotoxic in aquatic environments. Some anions such as [BF_4_]^−^ are also not stable as a result of the anions hydrolysis [11]. Other anions like trifluoroacetate ([TFA]^−^), bis(trifluoromethylsulfonyl)amide ([TFSA]^−^) and triflate are included in the first ILs generation. The second ILs generation, reported in 1992 by Wilkes and Zaworotko [14], relies on ILs with tunable physical-chemical properties, with high interest in developing materials for energy applications and metal ions complexation, allowing the development of functional materials. This generation of ILs also contains more biocompatible cations and anions derived from natural and eco-friendly products such as amino acids (AA) and carbohydrates. Their physical-chemical properties can be more tunable to specific functions (high energy density, chemical reactivity, electrochemical window, or chiral induction) [11]. The third generation of ILs is characterized by the development of ILs containing a biological property, and also with more specific chemical and physical properties for pharmaceutical (antibacterial, anticholinergic, local anesthetic, and antifungal agents) and biological applications [11]. As an example, the solubility capacity of ILs is astonishing given all the possible IL combinations (more than 10^18^ without considering double salts [15]) which allow tuning for hydrophobicity, number of hydrogen bonds, or in general for the necessary complementarity for good solubilization of the desired drug. Second and third IL generations are the most commonly used in biomedicine and also contain the most eco-friendly ILs since many can be derived from renewable sources, either of the cations or the anions. Thus, ILs with enhanced and tailor-made properties are being developed [11], also allowing the possibility of developing novel hybrid materials for more advanced uses.

ILs are considered non-toxic for manipulation since the ILs liquid state is not due to the presence of a solvent but rather due to the properties resulting from the specific combination of cations and anions. This fact, together with their tunability of polar/apolar moieties, leads to them being considered as solvents suitable for “green chemistry” applications [16,17]. Nevertheless, and despite being considered a green solvent, their long-term toxicity for biological systems, as well as their poor biodegradability are still a focus of concern [17]. Novel strategies for overcoming these issues are being developed. As an example, the toxicity of an IL containing a pyrrolidinium cation is significantly reduced when alkyl chains are substituted by oxygenated alkyl chains [18].

ILs are mainly derived from petroleum-based constituents such as imidazolium and pyridinium and can display toxicity and release hazardous decomposition products under certain conditions [19,20,21,22]. This has led to their “green” nature being questioned over the past few years [23,24]. Due to the strong socioeconomic impact of ILs and the doubts around their “green” nature, the synthesis of biocompatible ILs has gained special attention, expanding their applicability in different fields, particularly in the biomedical area.

The synthesis of novel ILs has been focused on bio-renewable and natural compounds such as amino acids and amino alcohols from proteins, sugars from cellulose, chitin starch, and other polysaccharides, aromatic aldehydes from lignin, and a diverse group of other compounds such as fatty acids from vegetable or algae-derived oils, among others [25]. To transform these biocompatible materials to IL cations and anions, a number of synthesis steps are required [25], and because of this, in some cases, it is difficult, considering the final biocompatibility of the resulting ILs.

**Figure 3:**
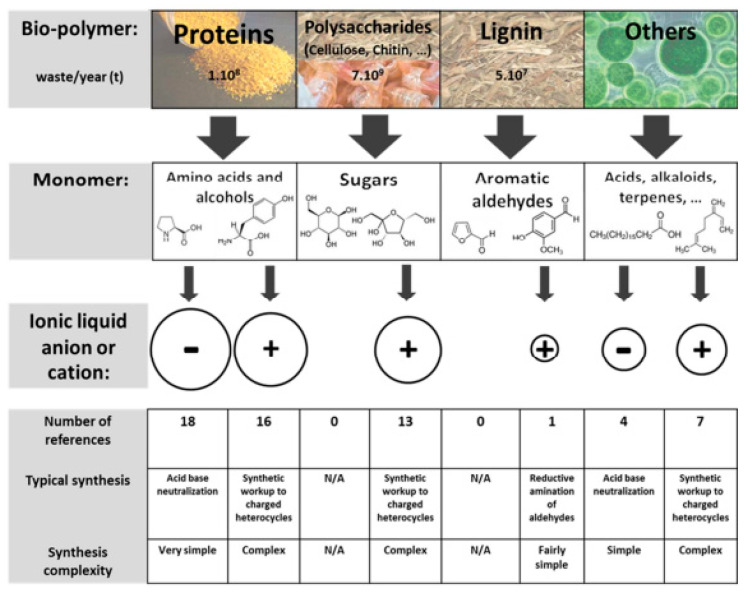
Synthesis pathways towards bio-based ILs. For each renewable resource class, typically derived monomers are indicated. The intensity of research (i.e., number of published papers) on converting these monomers into IL anions or cations is represented by the radius of the circle around the ‘−’ or ‘+’ sign, respectively. Typical synthetic routes and their complexity are mentioned where available. Reprinted with permission from [25].

The synthesis of biocompatible ILs has gained special attention due to their strong potential, with their synthesis mainly focused on protein-derived AA cations and anions, due to the non-toxicity, biodegradability, and biocompatibility of the derived purified protein compounds [25]. For instance, imidazolium room-temperature ILs (RTILs) cations have been combined with different natural AAs, allowing the synthesis of ILs with different functional anions. Another example are sugar-derived ILs, e.g., derived by the transformation of D-fructose monomers (isomannide, glucose, and glucose derivatives, namely arabinose and isosorbide) into monosubstituted imidazole compounds, that have been synthetized by the depolymerization or direct refining of polysaccharides [25]. The first polysaccharide-based RTIL was developed in 2007 being prepared through the combination of carboxymethylated chitosan and 1-ethyl-3-methylimidazolium hydroxide ([Emim][OH]). The use of aldehydes derived from lignin and hemicellulose [25] has also been reported. Currently, cholinium (Cho) and glycine-based ILs are considered the most commonly employed for biomedical applications (Figure 3) [25].

For biomedical applications, the toxicity (or biocompatibility) of ILs has been studied to evaluate their effect on the environment and humans with different cell lines [26,27,28,29,30]. The comparison between the commonly used ILs and novel ones validates the improved benign character of these compounds [28,31,32].

As previously mentioned, a large part of the research has been performed on the synthesis of biocompatible IL cations and anions from protein-derived amino acids [25,33]. Amino acids are non-toxic, biocompatible, and biodegradable chemical molecules [34], their being one of the most abundant organic compounds in nature increases their applicability for producing different classes of ILs [35,36]. The first synthesis of ILs in this scope was performed by Fukumoto et al. [37] from natural amino acids and 1-ethyl-3-methylimidazolium [Emim]^+^ as cation. Different room-temperature ILs have been developed since then due to their possible combination with different anion types, it being possible to analyze the effects of anion structure on the properties of the corresponding salts [37]. Further, new generation of ILs have also been developed in which the chiral cations are directly derived from naturally occurring α—amino acids and α—amino acid ester salts [36].

ILs originated from AAs have been described in the literature with an approximately equal number of references describing the synthesis of anions and cations to form ILs. In contrast, sugars resulting from the depolymerization of polysaccharides, or by direct refining, have only been converted to IL cations [25]. Fructose has been used as the starting material for the preparation of a new class of room-temperature ILs, being the first study related to the synthesis of ILs derived from naturally occurring sugars. These ionic liquids exhibit tunable solvent properties similar to conventional imidazole-based room temperature ILs [38] and a variety of studies have been reported with fructose derivatives as IL precursors [39,40,41,42]. The first natural oligosaccharide-derived room temperature ILs were prepared from [Emim][OH] and carboxymethylated chitosan by acid–base neutralization reaction [43]. Although efficient, imidazolium cations are expensive and thus limited in their large-scale industrial deployment. Regarding the replacement of imidazolium based ILs with those derived from renewable sources, studies have been reported based on aldehydes from lignin and hemicellulose. These compounds show significant potential for the realization of a process for future lignocellulosic biorefineries achieving economic impacts for other IL-based process technologies that currently use ILs synthesized from petroleum sources [44].

The most effective strategy for the development of biocompatible compounds relies on the use of choline as the cationic moiety in the structure. In this sense, the best candidate cation for the preparation of highly biocompatible amino acids ILs is choline, classified as vitamin B4 (also known as 2-hydroxyethyltrimethyl ammonium chloride), an essential micronutrient for cells. It is involved in many functions, including memory and muscle control, due to its function as a precursor for the neurotransmitter acetylcholine and also exists in the head groups of two classes of phospholipids that are abundant in cell membranes [45,46]. Its biocompatibility makes it an attractive cation for biomedical applications.

Several studies demonstrate that the use of this component as cation allows the development of biocompatible ILs with biodegradability and very low toxicity [47]; nonetheless, it has been reported [48] that more detailed toxicological tests with different organisms are necessary to truly determine the biocompatibility of the choline amino acid ILs. The combination of cholinium cations with other compounds such as amino acids, artificial sweeteners, or carboxylic acids such as anions has been performed in recent years [45].

ILs formed from active pharmaceutical ingredients have strong potential but have been poorly exploited [12]. Active pharmaceutical ingredients such as phenytoin, ampicillin, nalidixic acid, niflumic acid, pyrazinoic acid, and picolinic acid are generally recognized as safe materials and have been studied in combination with the cholinium cation [49,50,51]. IL strategies can take advantage of the dual nature of ILs to improve controlled solubility, bioavailability or bioactivity, stability, and for the elimination of polymorphism, allowing development of new delivery agents or customized pharmaceutical cocktails [12]. These developed active pharmaceutical ingredients based on biocompatible IL systems offer possibilities for advanced and improved treatment platforms.

Another alternative to develop biocompatible ILs relies on the use of anions derived from biological buffers (Good’s buffers) [52]. The buffer to be used in biological and biochemical studies must be chemically inert, should not interfere with metal ion-protein binding, must be non-toxic, should not absorb light in the UV-visible region, should be commercially available at a low cost, their pK_a_ values should be between 6 and 8 and should not vary with temperature, and must present high water solubility and low solubility in organic solvents [53]. The best buffers are zwitterionic amino acid derivatives, and they are the most widely used biological buffers, mainly due to their buffering capacity and low toxicity [53,54]. Due to the advantages of using cholinium as a cation and the background knowledge about Good’s buffers, it is reported in the literature that the synthesis of novel cholinium-sulfonate biocompatible ILs exhibit buffering properties [55]. As an example, the solubility of cytochrome c in hydrated choline buffers has been successfully achieved [55]. Considering the function of cytochrome c in cellular oxidation in plants and animals (as an electron-carrying mitochondrial protein), the strong biological impact of those findings leads to studies on new sets of biocompatible ILs from the same class [54,56].

Besides the large attention devoted in cholinium based ILs and the intensive research in this field, some biocompatible alternatives to cholinium as cation for biocompatible ILs synthesis have received consideration. In recent years, glycine betaine has received much attention. Glycine betaine is a natural and cost-effective substance possessing a quaternary trimethylalkylammonium moiety and a carboxylate function, useful for conceiving new ILs [57]. This compound is an abundant natural raw material that represents 27% of sugar molasses in beet by weight and is obtained after the extraction of saccharose [57]. A novel set of biocompatible ILs containing an esterified glycine-betaine as cationic moieties with different alkyl chains and different inorganic and organic anions has been also reported [57]. In all cases, the lengthening of the alkyl chain enhances the thermal stability of these ILs. The thermal characteristics and viscosity of these ILs with inorganic anions are different when compared to those with organic anions. Additionally, applying the interfacial properties to determine the possibility for these compounds to self-assemble has been addressed, as they could be used for solvent-based applications or in extraction processes [57,58]. Thus, biocompatible ILs from this family were used to remove pesticides from wastewater [58].

Glycine betaine has been recently combined with deep eutectic solvents, also known as deep eutectic mixtures or low melting mixtures, consisting of mixtures of choline halide–urea, choline chloride–thiourea, chloro–choline chloride–urea, and betaine hydrochloride–urea, being demonstrated to be an effective solvent for polymer dissolution and processing [59,60] due to their low cost, easy preparation, tunable physicochemical properties, negligible vapor pressure, non-toxicity, bio renewability, and biodegradability [61,62].

### 1.2. Ionic Liquids for the Development of Biomaterials

**Figure 4:**
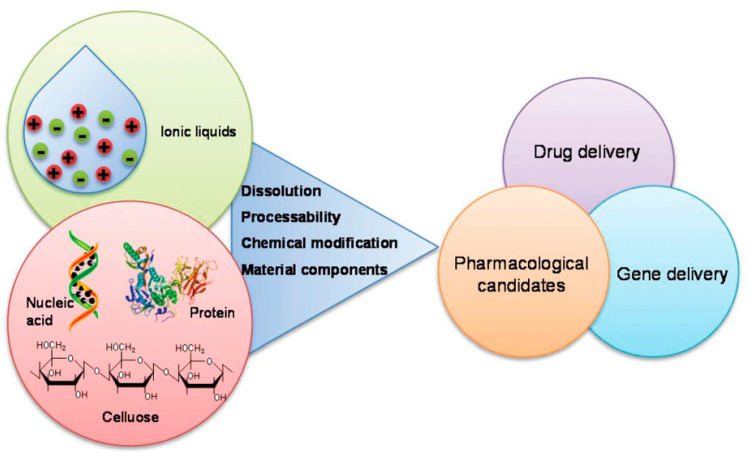
Strategies and application of IL/biopolymer materials. Reprinted with permission from [68].

The combination of ILs with different polymer-based materials has been explored in recent years. For the preparation of biomaterials, ILs can play different roles by assisting or participating in the formation of the materials through polymer dissolution or polymer regeneration. Typically, ILs have been used for the dissolution and processability of biopolymers and as reaction media and/or catalysts. The nanostructural organization of ILs [63,64] is in the form of aggregates (micelles) in aqueous solutions [65,66,67] or reverse micelles in nonaqueous systems [64]. IL/biopolymer hybrid materials are emerging as biomaterials in biomedical applications (Figure 4) [68]. Due to the large number of available combinations of ILs, and based on their intrinsic properties, they are being successfully implemented in a variety of applications in this field, such as biodegradable composite biomaterials [69,70,71], or pharmaceuticals [68,72], among others.

ILs have been also incorporated into different polymer matrixes to develop ionic polymer-based materials with strong application potential [1].

For the development of IL/polymer-based materials, a wide range of synthetic and natural polymers have been used such as poly(vinylidene fluoride) (PVDF) and its co-polymers, poly(L-lactic acid) (PLLA), silk fibroin, or cellulose, among others [1]. Among the different IL types, choline-based ILs are the most commonly employed in the biomedical field due to their enhanced biocompatibility and non-toxicity [73]. Additionally, choline cation is known as a phospholipids precursor comprising biological cell membranes in mammalian and plant tissues, being physiologically and environmentally decomposed in small molecules chains [73].

#### 1.2.1. Dissolution

ILs are commonly employed as solvents for polymer dissolution during biomaterials processing. For many biopolymers, ILs have shown great potential as solvents for most of the different polysaccharides, but also for DNA and proteins, due to their capacity to form intermolecular interactions such as dispersive, ionic, and dipolar interactions, as well as hydrogen bonds [68]. Different forms/morphologies can be obtained in this way, including films, porous scaffolds and structures, hydro- and areo-gels, nanocomposite gels, and micro and nanoparticles [74]. Among the most used polymers in IL dissolution are chitin, cellulose, and silk fibroin, being imidazolium-based generally employed in this process [68,70,74,75,76].

**Figure 5:**
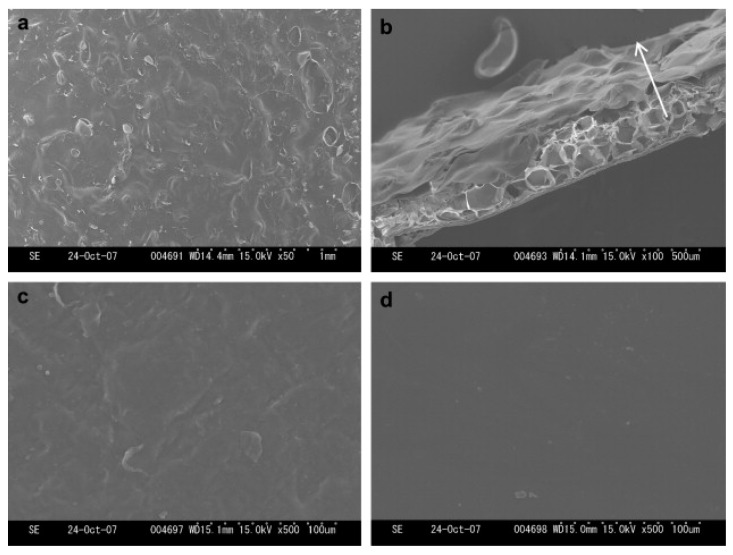
SEM micrographs of α-chitin samples obtained with [Bmim][Ac] as solvent: (**a**) surface and (**b**) cross-section of freeze-dried sponge, (**c**) surface of oven-dried film with methanol as anti-solvent, and (**d**) surface structure of oven-dried film with water as anti-solvent. Reprinted with permission from [78].

Chitin is the most commonly used biopolymer in IL dissolutions [70,71,77]. A typical chitin dissolution is an energy and waste-intensive multi-step process occurring at elevated temperatures, often producing degradation of the polymer during the process [77]. Imidazolium-based ILs such as 1-ethyl-3-methylimidazolium acetate ([Emim][Ac]), 1-butyl-3-methylimidazolium acetate ([Bmim][Ac]) and 1-allyl-3-methylimidazolium bromide ([Amim][Br]) have been used in the dissolution of this polymer, obtaining higher chitin concentrations and molecular mass and purity when compared to traditional processes [78,79]. Wu et. al., used [Bmim][Ac] for chitin dissolution, with posterior different drying methods of the samples. It was shown (Figure 5), that while the freeze-drying method originates a porous membrane structure, oven drying produces compact films, with a smoother surface using water as an anti-solvent [78].

Additionally, deacetylation into chitosan does not occur with the use of ILs [79]. Other ILs such as choline hexanoate ([Ch][Hex]) and choline citrate ([Ch][Cit]) have also been successful in this process [80].

Cellulose is a promising material for biotechnological applications as it has excellent biocompatibility and mechanical and thermal properties [76]. Several studies in the dissolution of monocrystalline cellulose have been performed, including studying the effect of the anion in 1-butyl-3-imidazolium ILs and the cation in acetate ILs in dissolving cellulose [81,82]. By studying the effect of the anion in the imidazolium-based ILs, it was concluded that the hydrogen bond-accepting strength was an important factor in polymer dissolution [81]. Also, by testing ILs with different anions it was concluded that while van der Waals interactions were not important for polymer dissolution, the presence of acid protons on the cations heterocyclic ring were essential in this process [82]. By complementing the use of ILs with techniques such as microwave radiation and ultrasonic process, an increase in the solubility of the ILs as well as in cellulose dissolution rate was found [83,84].

Another important material for biomedical applications is silk. Silk, widely used as a suture material, has also shown promising potential in tissue regeneration strategies due to its biocompatibility, mechanical properties, and slow degradation [74]. Chloride and acetate-based ILs have proven to be efficient in the dissolution of silk fibroin [85]. Additionally, amino acid-based ILs have proven to be good solvents by dissolving more than 20% of silk at 100 °C [86].

ILs have the ability to dissolve polymers in mild operating conditions and at atmospheric pressure [87]. However, as they need to diffuse completely in the molecular structure to disorder the polymer chains and disturb the ordered and amorphous regions, higher temperatures (typically ranging between 70 and 130 °C) can be necessary [88]. Polymer dissolution with ILs can be complemented with microwave radiation or sonication, reducing the dissolution time and the energy consumption when compared to higher temperature dissolutions [89,90]. However, this technique can lead to polymer degradation and/or incomplete dissolution [68].

Disruption of hydrogen bonding is highly dependent on the interactions between the ILs anion and the polymer functional groups (–NH_2_ and –OH). In halide ions, the chloride anion seems to present the highest efficiency in dissolving polymers, however, the molecular mass is often significantly reduced due to acidic hydrolysis [91]. A good alternative is the acetate anion, as it is less aggressive with respect to polymer degradation and has a lower viscosity, stronger basicity, and higher polarity for hydrogen bonding when compared to the chloride anion [92]. In addition, higher dissolved polymer concentration and lower dissolving temperature are also advantages with this anion [78].

For the same IL, dissolution efficiencies are different for different polymers. As an example, under the same conditions, by using the IL [Emim][Ac], cellulose can be easily dissolved up to a polymer content of 12 wt% whilst chitin is only dissolved up to a content of 4 wt% [93]. This is attributed, together with the different polymer chemistry, to the different polymeric structures, including polymorphic forms and amorphous regions, in which the dissolution of ordered structures is more difficult for amorphous regions. Furthermore, functional groups within the polymer molecules also affect dissolution [68].

Water absorption of ILs must be also considered in polymer dissolution, often being considered as an impurity or co-solvent, as it influences polymer solubility, especially in carbohydrates. In this particular case, water molecules influence the solvation of ILs by linking glycosidic units, leading to aggregation and thus, decreasing the polymer solubility to ILs [68]. While water decreases cellulose dissolution with [Emim][Ac], it represents a good co-solvent aiding for starch dissolution [94,95].

#### 1.2.2. Polymer Regeneration

Once a polymer is dissolved in an IL, it can be precipitated, i.e., regenerated, using a so-called anti-solvent such as water, ethanol, methanol, or acetone due to the polymers IL/anti-solvent low solubility. Thus, a so-called regenerated biopolymer is obtained presenting a rougher morphology when compared to the original polymer [96]. The anti-solvent must be carefully selected as it strongly influences the final properties of the materials. Polymer hydro- and organo-gels can be prepared by sol–gel transition using water and an organic solvent respectively as anti-solvents [97]. Dissolved chitin can be regenerated with the use of water, methanol, or ethanol [78,86]. Here, chitin regenerated with methanol as an anti-solvent shows lower crystallinity when compared to the one regenerated in water [78]. Anti-solvents such as water, methanol, ethanol, acetone, and acetonitrile have been also used to regenerate cellulose [74]. Water proved to be the most effective one as it effectively breaks the IL/polymer bonds, aiding the bonds between cellulose molecules [98]. On the other hand, the use of dimethyl sulfoxide (DMSO) and dimethylformamide (DMF) as anti-solvents in cellulose dissolved in [Bmim][Ac] has not been successful, as it did not make it possible to form films from the solutions [99]. Cellulose regenerated with water from a solution of [Emim][Ac]/DMSO resulted in a hydrogel with higher crystallinity and smaller pores than those regenerated from methanol [100]. In silk regeneration, methanol and acetonitrile have proven to be good anti-solvents, unlike water, due to the high solubility of silk both in the ILs and water. Acetonitrile regenerated silk resulted in a structure with lower crystallinity when compared to the one regenerated with methanol. Additionally, in the latter case, the produced films were transparent [101]. With respect to wet-spinning, only methanol has proven to be efficient as an anti-solvent in a silk solution with 1-ethyl-3-imidazolium chloride ([Emim][Cl]) [86].

#### 1.2.3. Preparation of Ionic-Liquid-Based Hybrid Materials

By using ILs, polymers can be processed into various forms including particles, fibers, films, scaffolds, or molded shapes. Posterior treatments such as polymer regeneration makes it possible to obtain hydrogels and the use of anti-solvents makes it possible to obtain organogels, which can be further processed into xerogel, cryogel, aerogel, or dense structures [74].

A polymer solution at room temperature can often form gel structures without the regenerating process, meaning that the IL acts as a component in the final gel material. These ionogels can later be used to form hydrogels when the dispersed phase is aqueous or organogels if the dispersed phase is an organic solvent [102]. The preparation of molded shapes from regenerated polymer hydrogels is a simple process, consisting in placing the polymeric solution into molds followed by a gelation process which occurs with immersion in an anti-solvent. However, care must be taken as hydrogels prepared with ILs tend to present heterogeneous segregated structures producing gel shrinkage during the regeneration process. Slow processing followed by a solvent exchange step is necessary in that case [103].

For application in the biomedical and environmental areas, polymer-based films and membranes are suitable structures. In these structures, typically, polymer hydrogel films are prepared by placing the solution into a mold or, in the case of a thin film, the solution is cast on a flat surface by rod- or spin-coating. The final film structures can be dried under several conditions such as low temperatures to obtain membranes and high temperatures or under vacuum to obtain compact films [104,105].

Fibrous structures are also interesting for biomedical applications, being mainly used as scaffolds due to their flexibility, high surface area, and high liquid retention and permeability. The main role of ILs in such structures relies on their use as solvents, being the fibers prepared by processes such as dry-jet wet-spinning and electrospinning [74,106]. Typically, the dry-jet wet-spinning process of a polymer solution containing ILs is performed by extrusion of the solution across an air gap into a coagulation bath. During the process of fiber formation, the IL is extracted from the fibers by the anti-solvent. The thickness of the fibers produced by this method is typically in the micrometer range. Different fibrous composites have been prepared by this method including cellulose/keratin and cellulose/chitosan fibers [107,108]. On the other hand, electrospinning enables the development of ultra-thin fibers [74] by using electric force to create a jet followed by stretching and elongation. The ILs high viscosity can be a disadvantage for this method. However, nanoscale nanofibers have already been produced with these materials with the help of cosolvents such as DMF or dimethylacetamide (DMAc). In such a process, ultra-thin fibers with good uniformity, higher crystallinity, and good thermal stability can be obtained [109].

Biopolymer/IL-based particles can be used in toxic chemical removal and enzyme support, among others [68,74]. Larger-sized particles, in the millimeter range, are typically prepared by a drop-wise method, where the polymeric solution is dropped into an antisolvent bath. In this method, the needle size, rate of dropping, IL and anti-solvent type, viscosity, and polymer concentration greatly influence the shape, size, and microstructure of the spheres [110]. The viscosity can be easily adjusted with the use of aprotic solvents [74]. On the other hand, micro-sized particles are prepared by the sol–gel method with an oil/polymer solution emulsion with the aid of a surfactant. The emulsion is mixed by ultrasonication and an antisolvent is used to regenerate the biopolymer. The type of anti-solvent and surfactant, polymer concentration, and oil/polymer solution ratio influence the size and shape of the particles and are also important parameters to take into account during the production of the particles [111].

Subsequently to the shaping of biopolymers into molded shapes, porous or compact films and particles, the biopolymer can be further modified into different dried forms, from highly dense homogeneous materials to low density porous foams, depending on the drying method [68]. Hydrogels and organogels can be changed to aerogels, cryogels, and xerogels by supercritical drying, freeze-drying, and ambient drying, respectively. They can also be modified by heating or vacuum drying to form a highly dense and voluminous shrinkage structure. These structures are of great importance for tissue engineering and controlled drug release [112].

ILs have been combined with different polymer matrixes to develop hybrid materials with different functionalities to be applied in different areas. In particular, for biomedical applications, ILs have been incorporated into different polymer matrices being processed into different morphologies and dimensionalities such as non-porous films, fibers, or 2D and 3D scaffolds. The major advantage of ILs in comparison with other polymer fillers relies on the presence of ionic charges able to promote in vitro cell adhesion and proliferation through the application of an electrical stimulus for cell stimulation.

**Figure 6:**
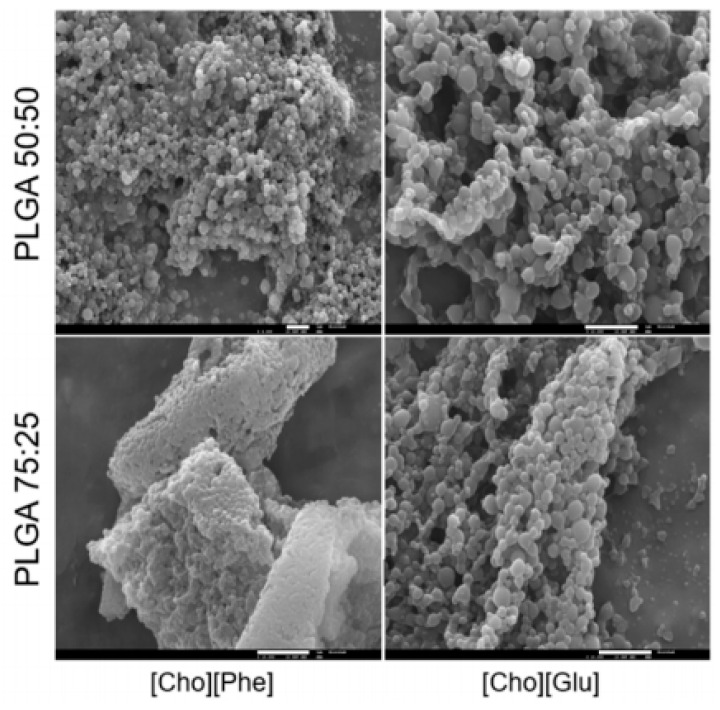
SEM micrographs of rutin-loaded IL-polymer nanoparticle hybrid systems after freeze drying at a magnification of 4000× (PLGA 50:50/[Cho][Phe] and 10,000× (remaining images). The scale bar of the micrographs at the bottom right of the images corresponds to 2 µm. Reprinted with permission from [114].

IL/polymer-based materials have been also processed into nanoparticles aiming for the development of hybrid systems for drug delivery. In this context, two choline-based ILs ((2-hydroxyethyl)-trimethylammonium-L-phenylalaninate ([Cho][Phe]) and (2-hydroxyethyl)-trimethylammonium-L-glutaminate ([Cho][Glu])) and poly (lactic-co-glycolic acid) (PLGA) 50:50 or PLGA 75:25 have been used to load rutin into the delivery system. The nanoparticles were produced by using a water in oil in water (W/O/W) double emulsion technique. According to this method, in the first step, the drug is dissolved in an aqueous solution (W_1_) and dispersed into the organic polymer solution (O), followed by mixture/homogenization. Then, the solution is dispersed in a second aqueous solution (W_2_), and the organic solvent is allowed to evaporate by stirring, the particles having formed [113]. The developed rutin-loaded IL-polymer nanoparticles present a diameter of 250–300 nm (Figure 6) and open a new paradigm for improving therapies of diseases treated with poorly soluble drugs.

**Figure 7:**
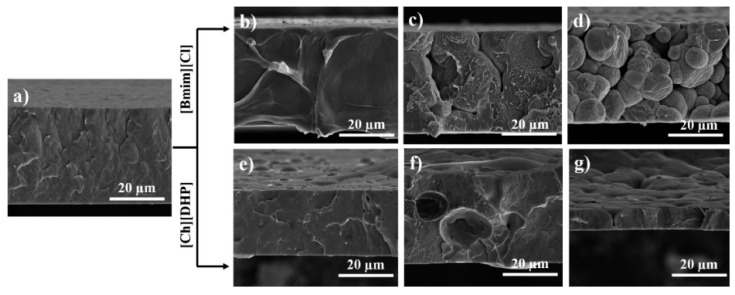
Cross section SEM images of (**a**) PVDF pristine and composite films with (**b**) 10% wt. [Bmim][Cl], (**c**) 20% wt. [Bmim][Cl], (**d**) 40 wt% [Bmim][Cl], (**e**) 10 wt% [Ch][DHP], (**f**) 20 wt% [Ch][DHP], and (**g**) 40 wt% [Ch][DHP]. Reprinted with permission from [114].

Different amounts of the ILs 1-butyl-3-methylimidazolium chloride ([Bmim][Cl]) and 2-hydroxyethyl-trimethylammonium dihydrogen phosphate ([Ch][DHP]) have been incorporated into poly(vinylidene fluoride) (PVDF) for the development of ionic electroactive materials in the form of films for muscle tissue engineering by a solvent casting method (Figure 7) [115]. This method allows the development of both porous and non-porous films, the solvent evaporation temperature being a critical step during material processing. Other studies report the potential applicability of IL/polymer films for biomedical applications [116]. 

**Figure 8:**
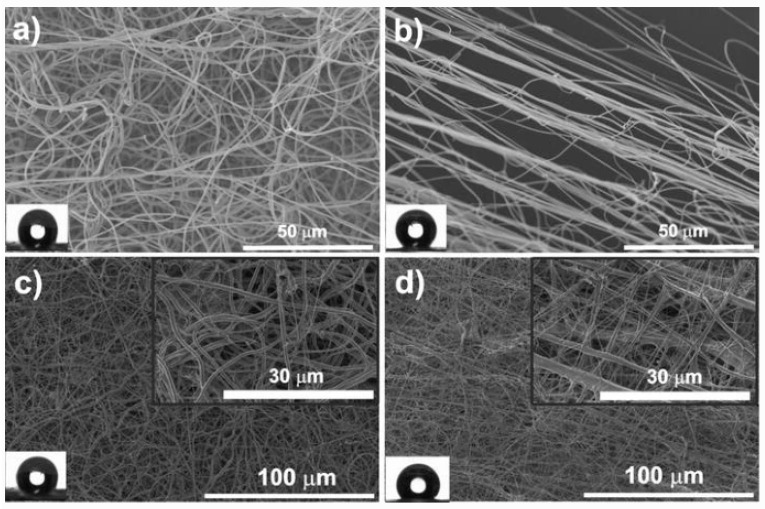
Morphology of electrospun polymer fiber mats: (**a**) randomly oriented neat PVDF, (**b**) aligned neat PVDF fibers, (**c**) randomly oriented PVDF with 5 wt% IL, and (**d**) aligned PVDF fibers with 5 wt% IL [Emim][TFSI] obtained. Reprinted with permission of [117].

Due to their high surface area, electrospun fibers also present strong potential for the development of IL/polymer-based materials for biomedical applications. PVDF comprising different contents of the IL 1-ethyl-3-methylimidazolium bis(trifluoromethylsulfonyl)imide ([Emim][TFSI]) were electrospun into random and aligned fibers for tissue engineering applications (Figure 8) [117].

## 2. Biomedical Applications of Ionic Liquids-Based Materials

### 2.1. Drug Delivery

**Figure 9:**
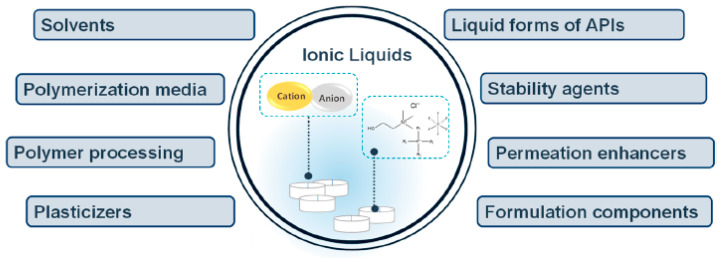
Applications of ILs in drug delivery systems design and development (APIs: active pharmaceuticals ingredients). Reprinted with permission from [118].

ILs have been used in drug delivery applications either to develop delivery systems and pharmaceutical formulations or to functionalize biopolymers (Figure 9).

Table 1 reports the main works using IL-based materials for drug delivery applications, presenting the used materials, the target application, as well as the main obtained results. 

**Figure 10:**
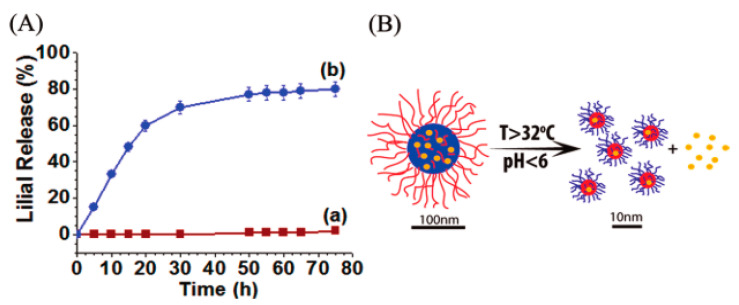
(**A**) Lilial release profile at simulated storage stage (a) and endosomal/lysosomal uptake stage (b): (a) 25 °C, pH = 7.4; (b) 37 °C, pH = 4.5. (**B**) Schematic representation of the dual responsive behavior of self-assembled PNIPAm-chitosan (CS)-Lilial nanocarriers. The PNIPAm and CS are represented in red and blue, respectively. Lilial is represented in yellow. Reprinted with permission from [127].

It is shown that the majority of the ILs are being used as green solvents to dissolve biopolymers [121,122,123]. For instance, [Bmim][Cl] has been used as the sole solvent to synthesize cellulose/chitosan/keratin composite materials [121]. Through this method, it is possible to obtain a composite that combines properties of each component: superior mechanical strength (from cellulose), hemostatic and bactericidal effect (from chitosan), and controlled drug release (from keratin). This composite material can be used, for instance, to treat chronic and ulcerous wounds. Conjugation of Lilial (a prototypical hydrophobic drug molecule frequently used in cosmetics) with chitosan has also been achieved using [Bmim][Cl] as a green solvent. The chitosan-lilial conjugate was further grafted with the thermoresponsive polymer poly(N-isopropylacrylamide) (PNIPAAm) in order to develop a temperature- and pH-sensitive drug delivery system for the controlled delivery of hydrophobic pharmaceuticals [127]. It was verified that PNIPAAm -cellulose- lilial copolymer conjugate self-assembles in nanocarriers with a size distribution that falls in the range 142 ± 60 nm and a smart-stimuli drug release profile suitable for intravenous administration of hydrophobic pharmaceuticals (Figure 10).

**Figure 11:**
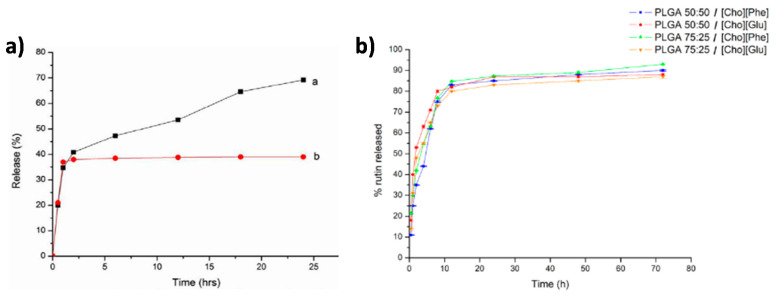
(**a**) In vitro release profile of a:5-FU loaded Ca- alginate/chitin nanofibers bio-nanocomposite gel beads and b: calcium alginate beads. Reprinted with permission from [122]. (**b**) Release profile of the rutin-loaded IL-PLGA nanoparticle hybrid systems during 72 h in phosphate buffer saline at pH 7.4. Data represented as mean ± SD (*n* = 3). Reprinted with permission from [114].

Imidazolium-based ILs (1-butyl-3-methylimidazolium hydrogen sulphate ([Bmim][HSO_4_]), 1-methylimidazolium hydrogen sulphate ([Hmim][HSO_4_]), choline-based bio-ILs (choline hydrogen sulphate ([Chol][HSO_4_]) and choline acrylate were used as solvents to synthesize chitin nanofibers, which were further used as doping to reinforce calcium alginate beads [120]. Besides enhancing the elasticity of the developed composite gel beads, it was verified that chitin nanofibers embedded calcium alginate gel beads retard the release of 5-fluorouracil release, an anti-cancer drug. As it can be seen in Figure 11a, while calcium alginate beads rapidly release 39% of 5-fluorouracil after 3 h at pH 7.4, calcium alginate/chitin nanofibers bio-nanocomposite gel beads show a sustained drug release profile, releasing about 70% of 5-fluorouracil after 24 h.

Aside from being used as alternative and green solvents to dissolve biopolymers, ILs are also being applied as one of the main components of the drug delivery carrier [114,116]. Aiming to improve the therapeutic effect of poorly soluble drugs, such as rutin, an IL-polymer nanoparticle hybrid system was developed [128] by combining PLGA with two different choline-based ILs: [Cho][Phe] and [Cho][Glu] [114]. Cytotoxicity assays showed that these hybrid systems are not toxic to HaCat human keratinocytes cells and it was verified that formulations containing [Cho][Phe] demonstrated significantly higher association efficiency values than formulations containing [Cho][Glu]. Both formulations presented a sustained drug release profile, indicating that the incorporation of the IL within the nanoparticles does not interfere with rutin release. The best formulation for the delivery of rutin was the IL-polymer nanoparticle hybrid system with [Cho][Phe] as IL, and with PLGA 50:50 as polymer (Figure 11b). Overall, these results demonstrate the applicability of the IL-polymer nanoparticle hybrid system as a valuable tool to deliver poorly soluble drugs. 

Chitosan films have been also loaded with two ammonium-based ILs (choline chloride ([Ch][Cl]) and [Ch][DHP] for developing electrically- and pH stimuli-responsive materials [116]. It was demonstrated that the incorporation of both ILs increased the electrical conductivity and the actuation capacity of chitosan films when stimulated by a low electrical charge, being this effect more pronounced for films loaded with [Ch][DHP]. A pH-sensitive drug release behavior was observed in [Ch][DHP]-based chitosan films. Regardless of the pH of the release medium, the amount of sodium phosphate dexamethasone (DXA) released from chitosan films doped with [Ch][DHP] was lower than that for non-doped chitosan films. Therefore, this simultaneous effect of the ILs on both conductivity and drug release profiles of the films allows the development of biocompatible and biodegradable drug delivery multi-responsive systems which can be used, for instance, for iontophoretic applications. 

Drug delivery systems have also been developed based on API, ILs, and polymers [124,125]. For instance, a drug delivery system of this type was synthesized [125] based on sparingly water-soluble mefenamic acid (MEF), ammonium ILs and grafted-poly(L-lactide) (MEF-IL-LA). Nanoparticles (NPs) of the MEF-IL-LA were formulated by the emulsion solvent evaporation technique showing that the NPs have spherical shapes and controlled sizes of 279 ± 3.6 nm to 453 ± 4.9 nm with maximum encapsulation efficiency up to 92.0 ± 2.7%. Moreover, controlled and sustained release up to 120 h and 168 h has been obtained, dependent on alkyl chain substituent and on the number of hydroxyethyl groups in the molecule, respectively.

### 2.2. Tissue Engineering

It is important to note that ILs based materials have been, until now, rarely used for tissue engineering (TE) applications, but due to their wide range of properties, they are starting to be explored as promising platforms for TE, namely for artificial muscles and for scaffolds, among others. In this sense, different kinds of approaches and material compositions containing ILs have been addressed. In particular, ionic electroactive films based on ILs and PVDF were developed [115] based on two different types of ILs, [Bmim][Cl] and [Ch][DHP], showing that their introduction in the polymer matrix induces the crystallization of the polar β-phase of PVDF and increases the electrical conductivity of the films. In both approaches, up to 20 wt% of IL, the proliferation of muscle cells was enhanced, demonstrating their applicability for muscle regeneration. In the same way, electroactive electrospun fiber composites based on PVDF and 1-ethyl-3-methylimidazolium bis(trifluoromethanesulfonyl)amide ([Emim][TFSI])—have been produced [117]. It was verified that this IL also induces the β-phase crystallization of the PVDF fibers, and that cell viability increases without influencing the morphology of muscle cells.

ILs have been used to dissolve different proteins, such as silk fibroin, keratin, and collagen to obtain scaffolds for TE applications. In particular, [Bmim][Cl] was used with silk fibroin to produce patterned films [129], and it was shown to support normal proliferation and differentiation of primary keratinocytes. In addition, the same IL was used to dissolve keratin, demonstrating that the migration of murine embryo fibroblast was faster when they were cultured in these hybrid scaffolds [130]. Regarding the dissolution of collagen, different kinds of ILs have been used. One of them was [Emim][Ac], whose biocompatibility was demonstrated with a fibroblast adhesion and proliferation model [131]. Triethanolamine acetate ([TEA][A]) was also used to dissolve collagen. After the dissolution, it was mixed with sodium alginate solution and hydroxyapatite to produce beads for the restoration of osteological defects [132], developing a system of active bone fillers for bone regeneration. In the same way, 1-butyl- imidazolium acetate ([Bmim][OAc]) was used to dissolve sucrose acetate isobutyrate (SAIB) and chitin in order to produce porous structures for TE and generating scaffolds with different porosities and with the absence of cytotoxic effect [133]. The dissolution of chitin and chitosan in ILs was also performed for blending with hydroxyapatite for bone TE applications [70]. 

For neural TE applications, [Emim][Ac] was used to dissolve chitin and form a uniform dispersion of CNTs [134], demonstrating that these composite scaffolds can be used as an implantable electrodes for the stimulation and repair of neurons. Beyond the dissolution of proteins, ILs were used to dissolve some polymers, such as cellulose using [Bmim][Cl] to obtain scaffolds with high porosity [135]. The produced scaffolds demonstrated absence of cytotoxicity, being a promising option to be used for TE applications. 1-n-allyl-3-methylimidazolium chloride ([Amim][Cl]) IL was also used as a solvent for cellulose for the production of cellulose scaffolds by the NaCl leaching method with bovine serum albumin (BSA) [136]. The biocompatibility and bioactivity of the scaffolds were demonstrated, as well the ability of mesenchymal stem cells (MSC) to attach to the surface of the produced scaffolds.

**Figure 12:**
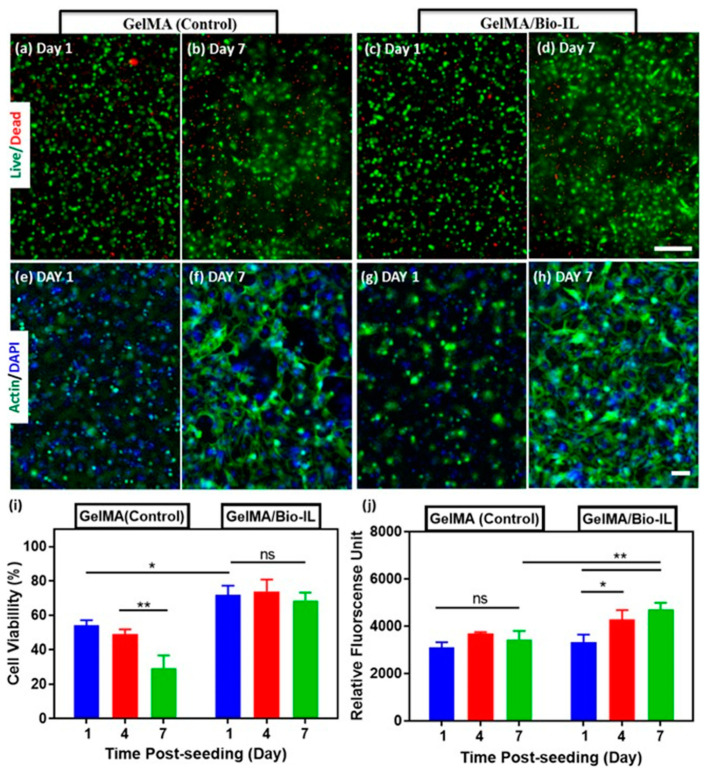
In vitro 3D encapsulation of cardiomyocytes (CMs) and cardiac fibroblasts (CFs) in GelMA/Bio-IL hydrogels. CMs and CFs (2:1 ratio) were 3D encapsulated inside visible light -rosslinked GelMA (control) and GelMA/Bio-IL hydrogels. Representative live/dead images from CMs/CFs encapsulated in GelMA (**a**,**b**) and GelMA/Bio-IL (**c**,**d**) hydrogels at days 1 and 7 post-encapsulation. Representative F-Actin/DAPI fluorescent images of CMs/CFs encapsulated in GelMA (**e**,**f**) and GelMA/Bio-IL (**g**,**h**) hydrogels, at days 1 and 7 post-encapsulation (scale bar = 200 μm). (**i**) Quantification of cell viability for 3D encapsulated CMs/CFs at days 1, 4, and 7 post-encapsulation. (**j**) Quantification of metabolic activity, relative fluorescence units (RFU), using PrestoBlue assay at days 1, 4, and 7 post-encapsulation. (* *p* < 0.05 and ** *p* < 0.01). All hydrogels were synthesized using 15% (*w*/*v*) final polymer concentration and 50/50 GelMA/Bio-IL ratio. Error bars indicate standard error of the means, asterisks mark significance levels of *p* < 0.05 (*) and *p* < 0.01 (**). Reproduced with permission from [73].

**Figure 13:**
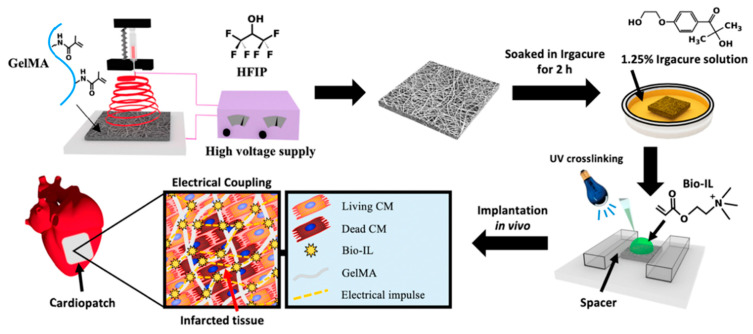
Synthesis and physical properties of electrospun GelMA/Bio-IL cardiopatches. Reproduced with permission from [140].

ILs were also used to process hydrogel-based constructs for TE applications, namely for cardiac tissue repair [73], skin regeneration [137], or implant applications [138,139]. GelMA/Bio-IL hydrogels were developed demonstrating that they can support the growth, and also perform in vitro 3D encapsulation, of primary cardiomyocytes and cardiac fibroblasts. For that, the spreading by F-actin/DAPI staining (Figure 12a–h) the cell viability (Figure 12i) and the metabolic activity (Figure 12j) were studied. It was verified that GelMA/Bio-IL hydrogels exhibited significantly higher viabilities comparing to the pure standard GelMA hydrogels. This kind of hydrogel was also used to develop conductive and adhesive cardiopatches [73]. The developed patches strongly adhere to the murine myocardium through the formation of an ionic bond between the tissue and the IL, removing in this way the need for suture (Figure 13). 

Promotion of the formation of chitosan/silk fibroin (CSF)/IL blended hydrogel systems for skin TE applications was also achieved using [Bmim][Ac]]. The in vitro studies revealed that these developed hydrogels can support the adhesion and growth of dermal fibroblasts. 1-vinyl-3-(3-aminopropyl)-imidazolium tetrafluoroborate ([VAPim][BF_4_]) and functionalized and non-releasing antibacterial konjac glucomannan (KGM) hydrogels have been also developed and used for the promotion of diabetic wound healing [140]. Further, a collagen-based hydrogel with [Emim][Ac] was developed for TE and cancer therapy [141]. The results showed that the spreading of the HepG2 and MKN45 cells (cancer cells) decreased more rapidly in these hydrogels than in the blank control, i.e., no hydrogel treatment.

Table 2 summarizes the potential applicability of ILs base hybrid materials in tissue engineering applications.

### 2.3. Cancer Therapy

The development of ILs plays an essential role in all aspects of the fighting against cancer. For instance, new developments in sensors for the detection of biomarkers at low concentrations have arisen. In the production of pharmacological drugs, it is used in their synthesis where it acts as a solvent facilitating their purification or allowing their extraction from natural products and enabling their stabilization. In drug delivery, it can act as a carrier, as an adjuvant, or directly as an active pharmacological ingredient (API) [3,143].

Given the unique properties of ILs acting as a green solvent, such as low volatility or high thermal stability, the first and more abundant use of ILs against cancer is in the solubility of drugs. Their use solves many problems associated with anticancer drugs. More than half of the anticancer drugs are in crystalline solid forms, which exhibit associated problems such as polymorphism and low bioavailability [4]. The good solubilization capabilities and IL physicochemical tuning have been used, for instance, to open new more complex routes for the delivery of the drugs such as topical delivery, which has become a promising route for treatment of external tumors such as melanoma [144].

The high number of possible ionic salts makes it also clear that ILs can be developed with a wide variety of well-tuned properties; therefore, making it possible to find good candidates with anticancer properties, i.e., acting as APIs (Table 3). It is also noteworthy that the initially described characteristics of ILs as green solvents are not so evident anymore, as their biological interactions have been evidenced in many studies. These effects, although they could be a disadvantage in environmental and some biotechnological areas, are very important in cancer therapies where cytotoxicity should be induced in cancer cells. In this regard, a broad body of experimental results has already been acquired for the most common ILs regarding their cytotoxicity towards several cancer cell lines [144]. For instance, the important role of hydrophobicity and lipophilicity of the cations has been determined [145,146]. It has been observed that an increase in the aliphatic side chain of imidazolium, ammonium, phosphonium, and pyridinium cations produces an increase in cytotoxicity [144], while the presence of oxygen on the side chain diminishes it [27,147]. This has been attributed to the interaction and insertion of the cation into the cell membrane [148,149]. Effects such as mitochondrial failure, oxidative stress, and apoptosis have been also observed in cancer cells [150,151,152]. In the case of therapeutic cancer treatment, the aim is to maximize the reported toxicity on cancer cells while minimizing the effect on healthy tissue. This contrast in cytotoxicity acting together with an enhanced permeation retention (EPR) effect is the basis of a successful therapy. Many encouraging results have shown a high dependence of the cytotoxicity with the cell type [153]. For instance, four ammonium and imidazolium ILs have been screened, 1-methylimidazolium chloride ([MIM][Cl]), [Bmim][Cl], triethylammonium hydrogen sulfate ([TEA][SO_4_H]), and triethylammonium hydrogen phosphate ([TEA][PO_4_H]), against brain cancer (T98G) and healthy human embryonic kidney (HEK) cells [154]. Interestingly, all the ILs showed less cytotoxicity against healthy cells, and cytotoxic effects on cancer cells above 0.01 mg/mL, [Bmim][Cl] being the one showing the highest anticancer activity.

Together with the use of more conventional ILs as APIs and thanks to their versatility, it is also possible to convert many drugs directly into an IL (API-IL). Egorava et al. described three ways to convert any drug into an IL [155]. Type I consists of the use of an ionic drug together with the oppositely charged ion, for instance with a cationic imidazolium and the anionic drug. Type II involves the covalent binding of the drug to the cation of the IL, also, although more rarely, to the anion. Finally, type III is a combination of both using two simultaneous drugs. In this work, they showed seven ILs containing salicylic acid. Together with an increase of solubility of salicylic acid, there was an increase of cytotoxicity on colorectal adenocarcinoma human cell line (CaCo-2) compared with conventional imidazolium-based ILs. Although in this case not much difference in toxicity was observed in healthy cells (fibroblasts 3215 LS), it opens the way to the preparation of more sophisticated drugs for cancer treatment.

**Figure 14:**
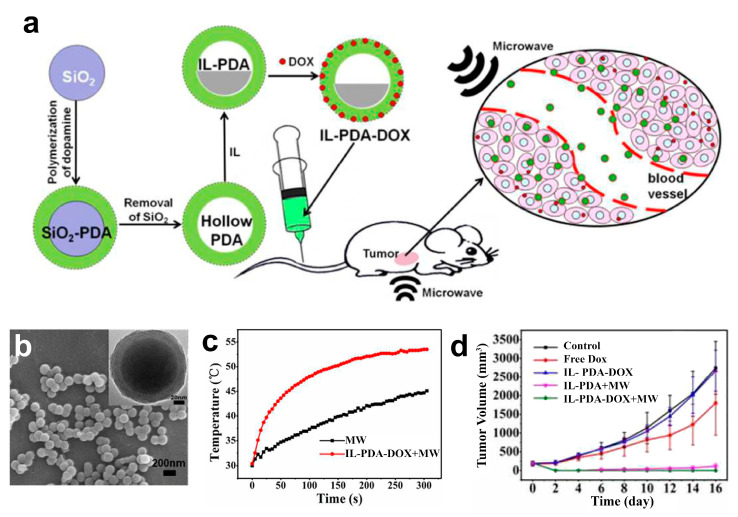
(**a**) Schematic illustration of the synthesis process of IL- PDA-DOX nanocomposites for combined chemotherapy and microwave-induced hyperthermia-therapy of cancer. (**b**) SEM and TEM images of IL-PDA-DOX nanocomposites. (**c**) Temperature rising curves of IL- PDA-DOX nanocomposites (1 mL, 10 mg/mL) irradiated with MW for 5 min. (**d**) In vivo chemotherapy and MW hyperthermia-therapy in ICR mice bearing H22 tumor (*n* = 4, per group). Change of tumor volume in each group. Reproduced with permission of the Royal Society of Chemistry [158].

A combination of ILs and other materials offers a higher degree of functionality in cancer treatments. For instance, several ILs have been combined with polymers to form a more sophisticated material for drug delivery systems. Cui et al. formed nanoparticles by complexing poly(ionic liquid-co-N-isopropylacrylamide) based on 1-ethylvinylpyridinium [EVPy] as the cation and deoxycholic acid [DA] as the anion [156]. The formed nanoparticles were used as drug carriers to transport the model drug doxorubicin (DOX) with a controlled delivery under changes in pH and temperature. Choline and polyacrylate have been also used to form nanogels for the delivery of 5-fluorouracil [157]. The IL system showed good stability and a prolonged drug release at stomach pH (1.2) for more than 10 days. In a different example, polydopamine nanoparticles loaded with [Emim][PF6] and DOX as model IL and anticancer drug respectively were used for combined chemotherapy and hyperthermia treatment. The system was used for the treatment of tumors in mice and, interestingly, the IL was used not to induce toxicity by itself but as a sensitizer for microwave radiation [158]. Figure 14 shows the formation of the generated nanoparticles using SiO_2_ as a sacrificial template for the formation of hollow polydopamine nanoparticles, which were subsequently loaded with the IL and DOX. The application of microwaves generates a local increase in temperature that induces cell death in the tumor region. The results showed complete removal of the tumor in 16 days while positive growth of tumors was observed for the rest of the controls (Figure 14d).

**Figure 15:**
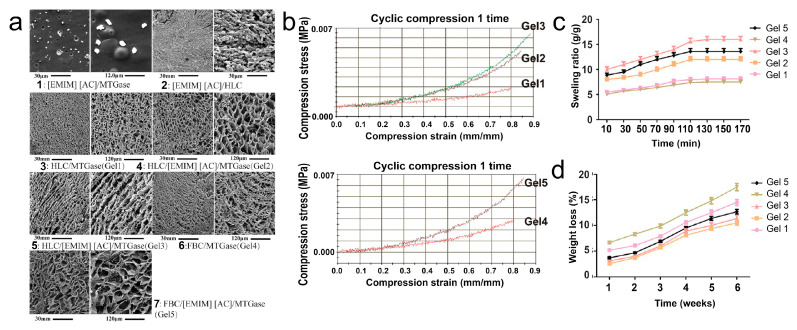
Comparative of three hydrogels of HLC and FBC at different incorporations of ILs. 0, 2, and 4 of mL_IL_ g_HLC_^−1^ for gel 1, 2, and 3 respectively, and 0 and 2 mL_IL_ g_FBC_^−1^ for gels 4 and 5 respectively. (**a**) SEM images of cross-sections of MTGase/[Emim][Ac] (1), HLC/[Emim][Ac] (2), Gel1 (3), Gel2 (4), Gel3 (5), Gel4 (6), and Gel5 (7). (**b**) Compression stress and compression strain of wet gels. (**c**) Swelling behavior in PBS solution (**d**). Degradation of hydrogels measured as the percentage weight loss in 6 weeks. Reproduced with permission of the American Chemical Society [141].

ILs have been also incorporated into biological hydrogels to improve their properties. Li et al. incorporated [Emim][Ac] in the formation of hydrogels of genetically engineered collagen (human-like collagen, HLC) and fish bone collagen (FBC) cross-linked by microbial transglutaminase (MTGase) [141]. The [Emim][Ac] had significant effects on the structure and properties of the hydrogels, including higher porosity, and mechanical strength (Figure 15). Moreover, the IL played an essential role in the inhibition of the enzymatic hydrolysis of the gel, which helped to maintain the physical functions for a longer time. Cell studies were performed on the gels using healthy fibroblasts 3T3-L1 and L929 cells and cancer HepG2 and MKN45 cells. Both HLC and FBC gels improved the fibroblast proliferation while they had a more inhibiting effect in the proliferation of cancer cells due to the anti-cancer properties of the IL and to the change of the structural properties of the gels.

The different IL and IL composites used for cancer therapies are summarized in Table 3.

### 2.4. Antimicrobial Agents

The ILs high surface activity is reported to be the main reason for their cytotoxicity. While this property is a concern for eukaryotic, mammalian cells, as it has been mentioned in the case of cancer therapies, it represents an interesting and important property in microbiology [163]. ILs have been reported to have the ability to interact with microbial cell walls in a mechanism that involves their aggregation into the membrane components, disrupting the cell membrane integrity [164], thus becoming a perfect biocide.

This feature is noteworthy since new materials/compounds able to destroy or inhibit the growth of bacteria and/or fungus is a worldwide necessity due to the emergence of bacterial resistance [165] and considering the challenging public health issue we are currently living in due to the new coronavirus SARS-CoV-2. These circumstances are a major threat to human lives and their successful management is of utmost importance. It is estimated that, on a global scale, antimicrobial resistance (AMR) may cause death to more than 10 million people annually by 2050, representing 45% of the total deaths [165]. Highly accounting for this problem are the overuse and misuse of antibiotics and the secondary infections caused by biofilms on indwelling medical devices, or due to viral or fungal infections [166].

One of the most attractive characteristics of ILs is the fact that they can be dissolved in a wide range of solvents, including water, which makes them suitable for biomedical applications. Aqueous solutions of certain ILs have been indeed reported to possess potent antimicrobial activity [167,168]. Although dissolved ionic liquids are not considered true ILs since they no longer consist exclusively of parent ions [169], this may indicate that the mechanism of action is due to one or both ions, both being able to possess inherent antimicrobial activity [170]. 

Early in-vitro studies on the relation between the structure of ILs and their potential antimicrobial properties have been performed on several microorganisms such as bacteria [171], earthworms [172], water fleas [31], zebrafish [173], and algae [174] and it has been stablished that the length of the cation side chain is the most significant indicator of biological activity. Long side chains of cation groups form a spatial heterogeneous region, aggregating in the membrane of microorganisms whose membranes are typically negatively charged [175]. Such characteristics make cations very reactive components towards microorganism’s cell walls, as proven by the observation of different physical phenomena of aggregation of different types of ILs with different cations at the surface of bacterial cells such as *Escherichia coli* [164,176].

In fact, antimicrobial IL solutions share a similar chemical structure to other well-established cationic biocides and surfactants, such as quaternary ammonium compounds, with both possessing characteristics such as charged hydrophilic head groups and hydrophobic tails [177]. Such similarities indicate that ILs may aggregate in solution to form amphiphilic micelles [177,178], which capacity is increased with increasing lipophilicity, which in turn may be manipulated by extending the substituent alkyl chain. Micelles are able to disrupt the integrity of the membrane, through electrostatic interaction between the cationic groups of the ILs and the anionic groups of the cell membrane, resulting in loss of the barrier function of the outer membrane [179]. This is the most widely accepted mechanism of action of ILs towards microorganisms such as bacteria, which is mainly attributed to the IL cation.

**Figure 16:**
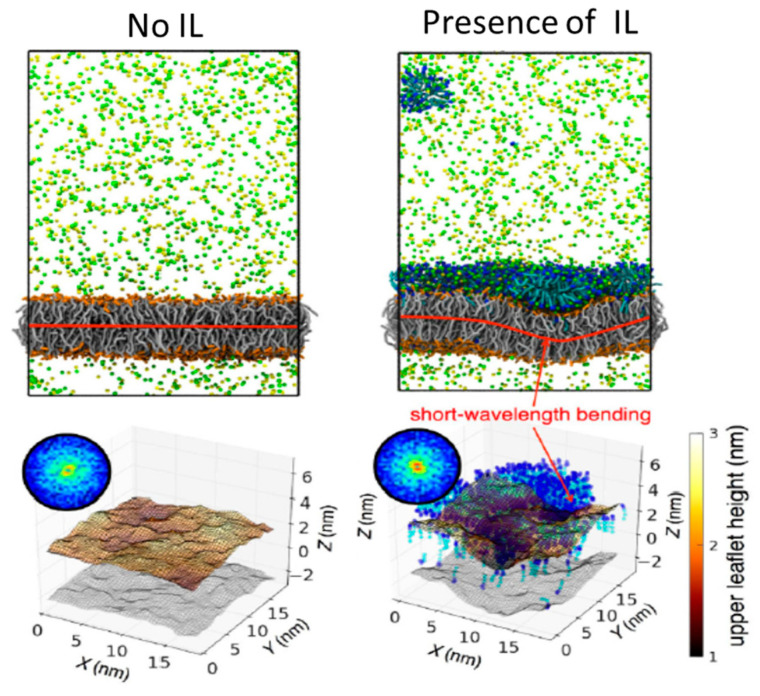
Upper row: snapshots of coarse-grained simulations of the capacity of ILs to induce bending of a 1-palmitoyl-2-oleoyl-sn-glycero-3-phosphocholine (POPC) bilayer lipid system with and without the IL, in this case 1-decyl-3-methylimidazolium chloride [DMIM][Cl]. Bottom view: Plotting of the 3-D contour surface of the upper leaflet height based on the phosphate group of the POPC. The bilayer center of the mass plane is shown in red to guide the eye. The lower leaflet surface is shown in gray. Spectral intensity based on the Fourier Intensity peaks corresponding to the bright red regions is associated with high amplitude bending modes of the bilayer surface. Adapted from [180].

Nevertheless, both cation and anion or the combination of both have been shown to interact with the membrane of the cell. Bioengineering simulation studies of the interaction of ILs at membrane model interfaces have demonstrated that either cations and anions are able to insert into a lipid bilayer, changing the structural and dynamic properties of the bilayer and leading to their permeability (Figure 16) [180]. A similar mechanism of action was observed when alkyltributylphosphonium chlorides were placed in contact with the fungus *Aspergillus nidulanms*, inducing damage on filaments and cell wall [181,182]. The potential toxicity of three alkyl [(1R,2S,5R)-(−)-menthoxymethyl] dimethylammonium chlorides has also been determined towards different Gram-positive bacteria (*Staphylococcus epidermis*, *Staphylococcus aureus*, among others) and Gram-negative bacteria (*Escherichia coli*, *Pseudomonas aeruginosa*) as well as wild type *Candida albicans*, also suggesting the effect of the increasing alkyl substituent chain length on their antimicrobial activity [183].

Apart of their direct effect on microorganisms, ILs antimicrobial activity can be boosted by combination with other common biocides, presenting an important recent research focus. For instance, the 1-alkyl-3-methyl imidazolium IL has been conjugated with silver and copper-containing anions to improve the antimicrobial activity against a range of pathogenic bacteria and fungi [170]. In another approach, an antibacterial ionic liquid and an antimicrobial peptide were conjugated via “click” chemistry, generating antimicrobial activity against Gram-negative multidrug-resistant clinical isolates, and antibiofilm action towards a resistant clinical *Klebsiella pneumoniae* isolate [184]. The introduction of organic salts from ciprofloxacin and norfloxacin as anions has been recently proven to enhance the antimicrobial activity against *K. pneumoniae S. aureus* and *Bacillus subtilis* strains [185]. Chitosan and lysozyme, well-known natural antimicrobials, have also been conjugated with different ILs to obtain a more potent antimicrobial agent. The guanylated chitosan derivatives showed a high antimicrobial activity in comparison with neat chitosan [186], while lysozyme was complexated with choline, lauryl sarcosinate, or deoxycholate and showed improved antimicrobial activity against Gram-negative bacteria *E. coli* and *P. aeruginosa* [187]. Also, the potential of ILs to improve the action of bioactive peptides has been recently shown. A covalent conjugate between an antibacterial IL and an antimicrobial peptide was produced via “click” chemistry. The resulting compound was found to maintain the peptide’s antimicrobial activity against Gram-negative bacteria multidrug-resistant clinical isolates as well as improved stability towards tyrosinase-mediated modifications [184].

**Figure 17:**
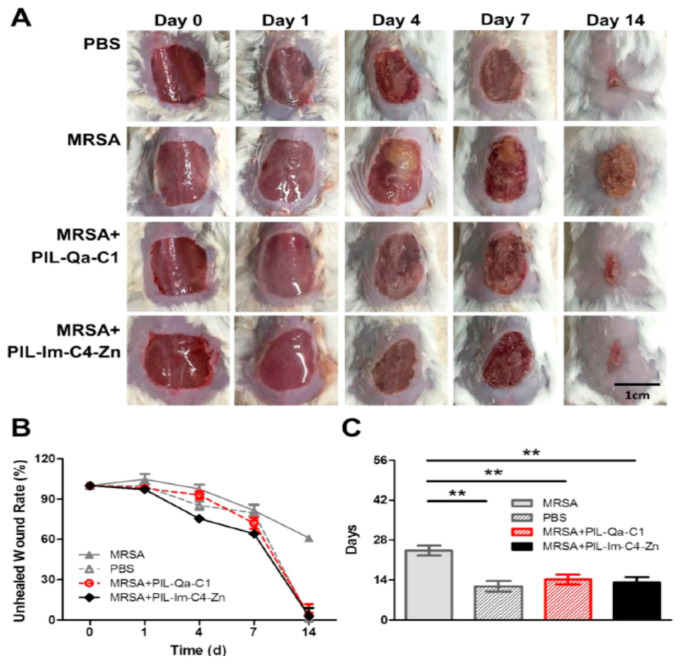
Wound healing evaluation of mouse model. Representative images (**A**) and unhealed wound rates (**B**) of skin wounds in 4 groups during 14 postoperative days. (**C**) Days for complete wound healing in four groups. Data are presented as mean ± SD, *n* = 6 in PBS negative control group and *n* = 11 in the other 3 groups; ** represents *p* < 0.01. Adapted from [189].

Materials and surfaces made of ILs have also been reported to avoid bacterial colonization and biofilm formation, which is an important feature for avoiding infections through contact with contaminated surfaces. Imidazolium-based membranes based on different cations and anions have shown antibacterial activity against *E. coli* and *S. aureus* which was more potent with increasing the cation chain length, at the same time being considered biocompatible [188]. Also, poly(vinyl chloride) substrates were processed with phosphonium ionic liquids as a coating to obtain a superhydrophilic surface that avoids the adhesion of *S. aureus* and *P. aeruginosa*, also possessing a bactericidal effect [188]. Quaternary ammonium and imidazolium-based poly(ionic liquid) membranes in the presence of zinc have also been produced and found effective for wound healing purposes in in vivo studies using methicillin-resistant S. aureus infected mouse (Figure 17) [189].

Despite that ILs have been shown to possess potent antimicrobial activity against different bacteria, including resistant strains, as well as fungus, it should be mentioned that most of them are also highly toxic towards mammalian cells. This is a drawback when such ILs are intended to be used for biomedical applications. For each antimicrobial IL studied, the cytotoxicity towards different cell lines should be also evaluated, determining the window of concentrations that are safe using.

Table 4 summarizes recent advances on ILs antimicrobial activity.

### 2.5. Biosensors and Biomedical Sensors

The importance of monitoring certain biomedical parameters and specific substances, especially in the field of environmental protection, health, safety, and industrial applications, has strongly increased in recent years. In fields such as medicine, accurate monitoring of certain substances can often mean the difference between life and death for a patient. Even though there have been many developments in the field of sensors, there is still a growing demand for systems that meet specific parameters and requirements, namely biocompatibility and biodegradability. These parameters are of paramount importance for biomedical devices. Therefore, biosensors, or sensors with both biological and physicochemical components that often meet the aforementioned parameters, have been gathering much interest in the last years.

**Figure 18:**
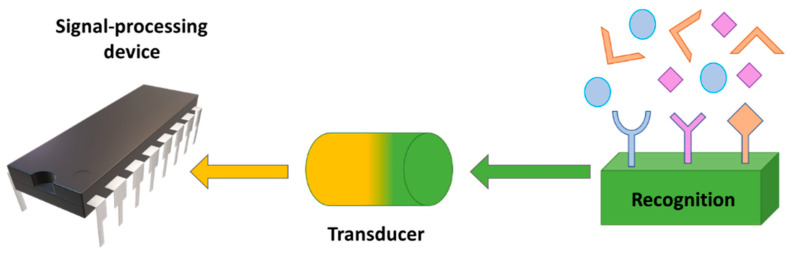
Schematic representation of the main elements of a generic biosensor.

In general, a biosensor has three main elements (Figure 18): a recognition element (such as enzymes or antibodies), a physical transducer (for example, electrochemical, piezoelectric, or thermometric) that converts the stimuli from the recognition element into a signal that can be processed, and a signal-processing device (light-sensitive or electronic) that translates this signal into something the user can comprehend [200]. Considering the inherent properties of ionic liquids and their ability to promote the stability of biomolecules, the evolution of the interest in these substances over the last decades becomes easy to understand [33].

**Figure 19:**
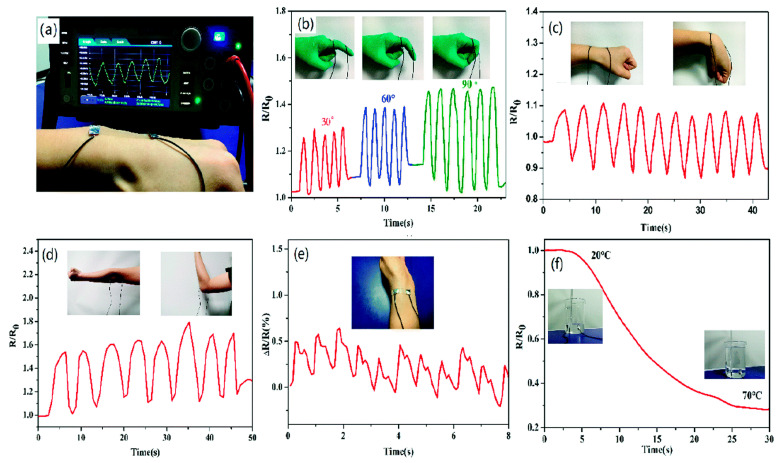
(**a**) Image of the testing of the ionogel strain sensor. (**b**–**d**) Relative resistance change of the ionogel for detecting different human motion: bending and straightening of the index finger, the wrist, and the elbow. (**e**) Relative resistance change of the ionogel for monitoring the beating of the human pulse. (**f**) Relative resistance change of the ionogel for monitoring the temperature change. Reprinted with permission from [204].

ILs have been growing in importance in the field of biosensors and sensors applied to the determination of relevant biomedical parameters, such as temperature, movement, position, force, or deformation, as different ILs change one or more of their properties as a response to external stimuli, such as electrical and magnetic fields, heat, or light [1,201]. As the specific functionalities of ILs can be tailored by the inclusion of functional groups, and they can be combined with many other materials, highly customizable sensors can be developed. One of the motives of the growing interest in them is that they could replace other, more toxic materials in applications where possible environmental impacts are a concern. In fact, one of the earliest commercial applications of ILs as sensors was in thermometers, by using ILs with thermometric properties, such as 1-butyl-3-methylimidazolium bis(trifluoromethanesulfonyl)imide ([Bmim][TFSI]) or 1-ethyl-3-methylimidazolium tetrafluoroborate ([Emim][BF_4_]) as a safer replacement for the commonly used mercury [202]. Other applications have also been found for [Bmim][TFSI], such as ammonia biosensing when combined with propylene carbonate [203], or in the development of an ionogel-based strain sensor for human motion monitoring (Figure 19) [204].

IL sensors and biosensors can also rely on optical responses, such as trihexyl(tetradecyl) phosphonium 2-(2-hydroxyphenyl) benzoxazole ([P_66614_][HBO]), which due to its fluorescence properties has been proposed as an SO_2_ gas sensor [205] and, more recently, for CO_2_ detection as well [206].

**Figure 20:**
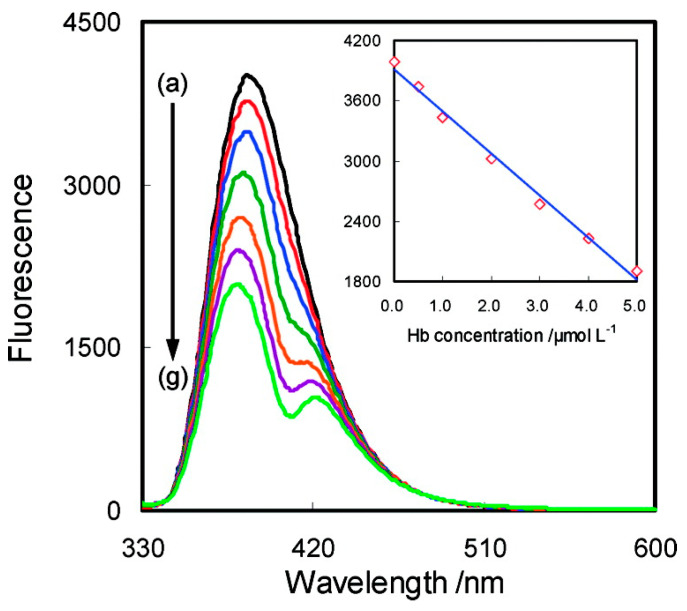
Fluorescence quenching of [BBim][Cl] by Hb (T = 293 K, λ_ex_ = 315 nm). Hb concentration (μmol L^−1^): (a) 0, (b) 0.5, (c) 1.0, (d) 2.0, (e) 3.0, (f) 4.0, (g) 5.0. [BBim][Cl] concentration in the aqueous solution: 0.01 mol L^−1^. Reprinted with permission from [207].

Use of [Bmim][Cl] in aqueous medium as a photochromic biosensor for hemoglobin (Hb) has been studied (Figure 20) [207]. Additionally, bis(1-butyl-3-methylimidazolium) tetrachloronickelate ([Bmim]_2_[NiCl_4_]) has been combined with PVDF for the development of thermochromic and thermoresistive sensors for temperature and humidity sensing [208] and different ILs have been studied for electrochemical detection of paracetamol [209].

**Figure 21:**
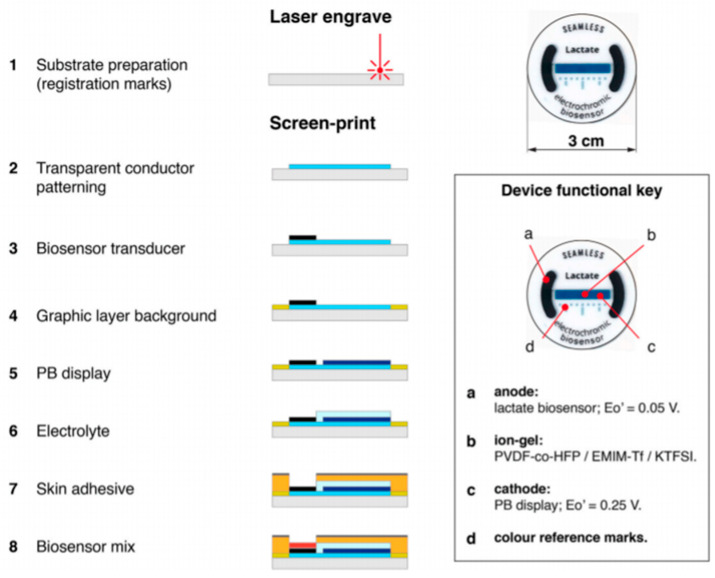
Diagrammatic representation of the fabrication process and photographs from two different prototype versions. The inset highlights the function of the device various components. Reprinted with permission from [210].

In order to be used in bioapplications, biosensors should be biocompatible and, if the application so requires, biodegradable as well. Their influence on biological processes should be either null or both positive and predictable, so as not to influence the measurements. For these applications, it is also desirable that these devices are self-powered, meaning that they do not require any external power source to function. A recent example of such a self-powered sensor was the development an ion-gel based poly(vinylidene fluoride-co-hexafluoropropylene) (PVDF-HFP) paired with the IL 1-ethyl-3-methylimidazolium trifluoromethanesulfonate ([Emim][TF]) for the electrochromic biosensing of lactate from sweat (Figure 21) [210].

The use of IL and IL hybrid materials for biomedical and biosensing applications are summarized in Table 5.

In biosensors, ILs often take the role of binders in the surface of the recognition element, such as in electrochemical biosensors. Their electrochemical properties allow them to immobilize the biological analytes on the surface of the electrode, allowing for this new generation of biosensing devices to deliver very precise measurements with extremely low detection limits [219]. This can be achieved by one of two processes: a) by the imbibition of the specifically designed and synthesized polymeric compound in the ionic liquid for the specific application, b) by the formation of a polymer-IL composite in situ by reaction-induced phase separation [220]. Each of these processes has advantages and disadvantages. The imbibition process is simple but does not allow for the phase interactions of the IL and polymer to be fine-tuned to the desired parameters. On the other hand, phase-separation allows much control over the physicochemical properties of the composite material and allows for various shapes and sizes to be fabricated—but it cannot guarantee that no unreacted monomers remain after the reaction is complete.

In addition to their ability to trap and immobilize analytes, ILs also display high sensitivity, selectivity, and reusability [221]. Furthermore, they are also often used as a non-volatile electrolyte in sensors that require them.

## 3. Main Conclusions and Future Trends

Due to their specific properties, ILs have been explored for a wide range of applications, of which biomedical applications are of particular interest and current activity. The large possible combinations of different cations and anions allow the development of a high and vast number of ILs with specific and tailored properties. Particularly for biomedical applications, the development of biocompatible and non-toxic ILs has been essential. Currently, the most commonly used ILs remain protein-derived amino-acids cations and anions and choline cations, but also other types are emerging in fields such as cancer treatment, biocides, or biosensing.

Besides the increasing interest devoted to ILs for different fields and application areas, the most common use of ILs is as green solvents for a wide range of polymers and other materials. Nevertheless, their combination with polymers matrixes to form IL/polymer-based hybrid materials is rapidly getting recognition in areas such as sensors, actuators, and tissue engineering where they have demonstrated an outstanding potential. Particularly for biomedical applications, and based on their different actuation possibilities, ILs are emerging as highly valuable candidates. Novel studies are demonstrating, for instance, the potential of ILs in drug delivery systems with photo-, temperature- or pH-sensitive drug release behavior. For tissue engineering, ILs have been employed as polymer solvents, and have been combined with polymers to develop a wide variety of responsive and functional materials, including films, fibers, spheres, membranes, and hydrogels. These early works make also evident that significant efforts to understand these systems are still necessary. For instance, the IL stability and release, in the case of drug delivery systems, needs to be better understood, while a lack of studies exists concerning the influence of the IL on the cell behavior of a larger variety of cells, namely their adhesion, proliferation, and differentiation. The effect of the ionic charges in the cells is also absent in many studies, reflecting the necessity of new works on the dynamics of the effect of the ILs ionic charges on cells.

The demonstration that certain ILs present less cytotoxicity against healthy cells than against cancer cells supports the potential of ILs to be used in the development of novel strategies for cancer therapies. Also highly related, the strong potential of ILs as antimicrobial and antifungal agents in biomedical applications, such as wound dressing, has been also demonstrated. In this field of applicability, advances are necessary to evaluate the cytotoxicity towards different cell lines determining the window of concentrations that are safe using. Finally, ILs have been also explored in the field of biomedical sensing and biosensing through the development of sensors able to recognize temperature and force variations, as well as to identify biomolecules, proteins, and pharmaceuticals, among others.

Despite the growing number of studies concerning the use of ILs in different areas of the biomedical field, strong efforts concerning the ILs toxicity, stability over time, and degradability as well the IL degradation products should be addressed deeply. Additionally, it is noteworthy that currently, a high number of studies report the use of ILs as solvents during preparation of materials, with still a limited number of studies existing on IL/polymer-based materials for biomedical applications. In this scope, the combination ILs with other materials allows them to confer interesting functional responses to the systems, including optical, catalytic, or shape memory. As an example, the exploration of the magnetic properties of ILs and magnetic ILs/polymer materials for tissue regeneration holds interesting potential. Different studies reports that the magnetic field can promote cell proliferation and differentiation mostly based on magnetic nanoparticles, which are toxic in certain concentrations. In this regard, magnetic ILs are of great interest due to the possibility of developing magnetic-particle-free magnetically responsive hybrid materials. Thus, IL and their tailorable properties as well as their rich synergetic effect in their combinations with polymers and related materials holds great promise as a next generation of smart and multifunctional materials for biomedical and biotechnological applications.

## Figures and Tables

**Table 1:** IL-based hybrid materials for drug delivery applications.

Materials	Ionic Liquids (ILs)	Biomedical Property	Ref.
Chitosan	[Ch][Cl]	Electrical and pH-sensitive drug delivery	[116]
	[Ch][DHP]		
Chitin	[C_2_mim][Ac]	Topical release drug delivery	[119]
	[Bmim][HSO_4_]	Sustained drug release application	[120]
	[Hmim][HSO_4_]		
	[Chol][HSO_4_]		
	Choline acrylate		
	Choline chloride-thiourea		
Cellulose/Chitosan/Keratin	[Bmim][Cl]	Bandage to treat chronic and ulcerous wounds	[121]
Cellulose/Fe_3_O_4_ NPs/Heparin	[Emim][Ac]	Magnetically responsive drug delivery	[122]
Locust bean gum	[Bmim][Cl]	Potential drug delivery system	[123]
	[Emim][Ac]		
	[C_2_OHmim][Cl]		
Active pharmaceutical ingredient/grafted-PLLA	Choline chloride	Potential drug delivery system	[124]
	di(2-hydroxyethyl)dimethyl ammonium bromide		
Active pharmaceutical ingredient/grafted-PLLA	2-hydroxyethyl triethyl ammonium bromide	Drug delivery system	[125]
	2-hydroxyethyl tributyl ammonium bromide		
	di(2-hydroxyethyl)dibutyl ammonium bromide		
	tris(2-hydroxyethyl)butyl ammonium bromide		
pH-sensitive polymer/montmorillonite (MMT)	3-methyl-1-[2-(2-methyl-acryloxy)ethyl]imidazolium chloride	Colon Specific Drug Delivery System	[126]
Cellulose/PNIPAAm	[Bmim][Cl]	Temperature and pH-sensitive drug delivery	[127]

**Table 2:** IL/polymer composites for tissue engineering.

IL	Polymer	Cells	Applications	Ref.
[Bmim][Cl]	PVDF	C2C12 cells	Skeletal muscle	[115]
	Silk fibroin	Primary keratinocytes	Tissue engineering scaffold	[129]
	Keratin	Murine embryo fibroblast	Tissue engineering matrices	[130]
	Cellulose	Human skin fibroblasts	Skin	[135]
[Ch][DHP]	PVDF	C2C12 cells	Skeletal muscle	[115]
[C_2_mim][NTf_2_]	PVDF	C2C12 cells	Skeletal muscle	[117]
[EMIM][Ac]	Collagen	Primary fibroblast	Tissue engineering scaffold	[131]
	Chitin	Cortical neurons	Neural	[134]
	Collagen-based hydrogel	HepG2 and MKN45 cells	Cancer therapy	[141]
[TEA][A]	Collagen-alginate-HA	Rat mesenchymal stem cells (rMSC)	Bone regeneration	[132]
[Bmim][OAc]	SAIB and chitin	Human adipose-derived stem cells (hASCs)	Tissue engineering scaffolding	[133]
[Amim][Cl]	Cellulose	Mesenchymal stem cells	Tissue engineering scaffold	[136]
Choline-based bio-ionic liquid (Bio-IL)	GelMA hydrogel	Co-cultures of primary cardiomyocytes and cardiac fibroblasts	Cardiac tissue repair	[73]
		Primary rats cardiomyocytes	Cardiac tissue repair	[140]
[Bmim][Ac]	Chitosan/silk-based hydrogels	Human dermal fibroblasts	Skin tissue engineering	[137]
[VAPim][BF_4_]	KGM hydrogels	L929 cells	Diabetic wound healing	[142]

**Table 3:** IL and IL hybrid materials approaches evaluated for cancer therapy.

IL	Material or Material/Drug	Cancer Type/Cell Lines	Ref.
[Emim][Sal], [Bmim][Sal], [Hmim][Sal]; ([Emim-OSal][Cl], [Prmim-OSal][Cl], [Emim-OSal][BF4]; [Emim-OSal][Sal]	Salicilic acid	Colorectal adenocarcinoma/human cell line (CaCo-2), CaCo-2 (colorectal adenocarcinoma) and 3215 LS (normal fibroblasts)	[155]
[EVPy][DA]	Poly(ionic liquid-co-N-isopropylacrylamide)/doxorubicin	Breat cancer	[156]
[Chol-A][5-FU]	Polyacrylate/5-flurouracil (5-FU)	Stomach cancer	[157]
[Emim][PF_6_]	Polydopamine/doxorubicin	Liver cancer/H22 tumor cell lines and HepG2 cells	[158,159]
[Emim][Ac]	Human-like and Fish bone collagen hydrogels	Liver and stomach cancer/healthy fibroblasts 3T3-L1 and L929 cells and cancer HepG2 and MKN45 cells	[141]
Choline formate	PHEMA/Curcumin	Ovarian Cancer/SKOV-3 cells	[160]
Cetpyrsal	Paclitaxel (PTX)	Ovarian and breast and pancreatic Cancer	[161]
[VHim][NTf_2_]	hyaluronic acid grafted poly(ionic liquid)/doxorubicin	Breast and colorectal carcinoma/MCF-7, CT26	[162]

**Table 4:** Antimicrobial ionic liquids and their effect on microorganisms.

IL		Microorganisms Tested	Effect	Ref.
Cation	Anion			
1-alkyl-3-methyl imidazolium	Chloride	*S. aureus* ATCC 29213	Broad spectrum antibiofilm activity	[167]
		MRSA (clinical strain 201)		
		Epidemic MRSA strain E-MRSA 15		
		*S.epidermidis* ATCC 35984		
		*S. epidermidis* ATCC 12228		
		*E. coli* NCTC 8196		
		*P. aeruginosa* PA01		
		*K. aerogenes* NCTC 7427		
		*Burkholderia cenocepacia* J2315		
		*Proteus mirabilis* NCTC 12442		
		*Candida tropicalis* NCTC 7393		
1-alkylquinolinium	Bromide	*S. epidermidis* ATCC 12228	Excellent, broad spectrum antibacterial and antifungal activity in both the planktonic and biofilm mode of growth.	[168]
		*Methicillin resistant S. epidermidis* (MRSE) ATCC 35984		
		*S. aureus* ATCC 29213		
		*Methicillin resistant S. aureus* (MRSA) ATCC 43300		
		*E. coli* NCTC 8196,		
		*K. aerogenes* NCTC7427		
		*Bacillus cereus* NCTC 2599,		
		*P. mirabilis* NCTC 12442,		
		*P. aeruginosa* PA01		
		*C. tropicalis* NCTC 7393		
Imidazolium-based	Iodide	*B. subtilis* 168	Antibacterial activity: the silylalkyl group is useful to generate antibacterial activity to imidazole salts	[190]
		*S. aureus* subsp., *aureus* NBRC 15035		
Pyrrolidinium-based		*E. coli* MG1655,		
Piperidinium-based		*P. putida* NBRC 14164		
1-alkyl-3-methylimidazolium	[AgX_2_] [CuX_4_]^2^	MRSA	An enhancement of overall IL antimicrobial activity is observed when anions and cations are both inherently antimicrobial	[170]
		MRSE		
Imidazo[1,5-a]quinoxalin-4-on-1-yl)-1-pyridinium	Bromide	*P. aeruginosa* 9027	Antimicrobial activity and selectivity towards Gram-positive bacteria and yeasts	[191]
		*E. coli* F-50		
		*S. aureus* 209p		
		*B. cereus* 8035		
		*Aspergillus niger* BKMF-1119		
		*Trichophyton mentagrophytes*		
		*C. albicans* 885-653		
Imidazolium-based with different alkyl chain lengths	Bromide	*S. aureus* ATCC 6538	The ILs’ antibacterial activities were improved with the increase of the alkyl chain length and higher charge density of imidazolium cations	[188]
		*E. coli* ATCC 8099		
Imidazolium-based Pyrrolidinonium-based	Cloride Bromide	*B. subtilis* KCTC1914,	Antibacterial and antifungal properties that improve with longer alkyl chains	[192]
		*S. aureus* 209 KCTC1916		
		*S. aureus* R209 KCTC1928		
		*E. coli* KCTC1924,		
		*S. typhimurium* KCTC1926		
		*C. albicans* KCTC1940		
		*Chllolella regularis* (algal bacterium)		
1-methyl-3-dodecylimidazolium;	Bromide	*E. coli* ATCC 25922	Antimicrobial activity and relatively low hemolytic activity	[193]
		*P. aeruginosa* ATCC 27853		
1-dodecyl-methylpyrrolidinium;		*S. epidermidis* ATCC 35984		
1-dodecyl-1-methylpiperidinium		*S. aureus* ATCC 6538		
		*Enterococcus faecalis* ATCC 29212		
Imidazolium-based Pyridinium-based	Bromide	*B. subtilis* ATCC6633	The conjugation of ILs with the acid-based anionic surfactant sodium N-lauroyl sarcosinate result in a strong synergism, resulting in enhanced interfacial and aggregation properties. Broad-spectrum antimicrobial activity against bacteria and fungus	[194]
		*S. aureus* ATCC29213		
		*S. epidermidis* ATCC12228		
		*Listeria monocytogenes* ATCC15313		
		*E. coli* ATCC25922		
		*Acinetobacter baumannii* ATCC19606		
		*P. aeruginosa* ATCC27853		
		*C. albicans* ATCC10231		
Imidazolium-based	Pyrithione	*L. monocytogenes* ATCC 13932	Incorporating pyrithione into an IL resulted in an improvement bactericidal effect, especially against gram-negative bacteria and a high susceptibility against clinically relevant yeast *C. tropicalis*	[195]
		*B. cereus* ATCC 1177		
		*S. aureus* ATCC 6538		
Trioctylmethyl- based		*E. faecalis* ATCC 19433		
Trimethyl-based		*Lactobacillus sakei* ATCC 15521		
		*Lactococcus lactis* ATCC 19435		
		*Geobacillus stearothermophilus* ATCC 7953		
		*Salmonella enterica ser. Typhimurium* ATCC 14028	Antibacterial, antiviral and antifungal properties	
		*E. coli* ATCC 25922		
		*Citrobacter freundii* ATCC 43864		
		*P. mirabilis* ATCC 29,906		
		*P. aeruginosa* ATCC 27853		
		*Saccharomyces cerevisiae* ATCC 4,000,850		
		*C. tropicalis* ATCC 750		
Imidazolium-based	Chloride	*S. aureus*	Antimicrobial activity against resistant Gram-positive and Gram-negative bacteria	[196]
	Bromide	*B. subtilis*		
		*K. pneumonia*		
		*E. coli* MC4100		
		*E. coli* XL-1 Blue		
Ammonium salt Phosphonium salt	Chloride	*E. faecium* 20,477	Broad spectrum of activities towards Gram-positive and Gram-negative. High cytotoxicity towards human cells	[197]
		*S. aureus* CIP 7625		
		*K. pneumonia* CIP 82.91		
		*A. Baumannii* ATCC 19606		
		*P. aeruginosa* 100,720		
	Bromide	*E. aerogenes* ATCC 13048		
	Iodide	*E. coli* CIP 54.8		
Imidazolium-based	Bromide	*S. aureus*	The bioactive peptide 3.1-PP4	[184]
		*E. faecali*	(KKLLKWLLKLLKTTKS, C-terminal amide) preserve its potent antibacterial and antibiofilm action, while significantly improving its stability towards an enzyme that is relevant in the skin wound environment	
		*P. aeruginosa*		
		*E. coli*		
		*K. pneumoniae* (clinical isolate)		
Choline;	Ciprofloxacin Norfloxacin	*K. pneumoniae*	The ILs were not toxic to healthy cell lines he antimicrobial activity against *K. pneumoniae* was particularly enhanced for the ciprofloxacin-based OSILs	[185]
1-ethyl-3-methylimidazolium;				
1-hydroxy-ethyl-3-methylimidazolium;		*S. aureus*		
1-(2-hydroxyethyl)-2,3-dimethylimidazolium;				
1-(2-methoxyethyl)-3-methylimidazolium;		*B. subtilis*		
acetylpyridinium				
Choline	Sarcosinate	*E. coli*	Lysozyme complexed with ILs, enhanced their antimicrobial activity	[187]
	Deoxycholate	*P. aeruginosa*,		
		*Bacillus thuringiensis*		
Phosphonium-based	Docusate (AOT)	*P. aeruginosa*	Development of poly(vinyl chloride) materials blended with phosphonium ionic liquids created a slippery, superhydrophilic surface, creating an antifouling surface	[198]
		*S. aureus*		
Alkyl[(1R,2S,5R)-(−)-menthoxymethyl]dimethylammonium (C_10_-Am-Men)	Chloride	*C. albicans*	Antifungal activity	[199]

**Table 5:** IL-based biosensing materials for biomedical applications.

IL	Polymer	Application	Ref.
[Bmim][TFSI]	PBA ionogel	Human motion sensing/monitoring	[204]
	Propylene Carbonate	Ammonia biosensor	[203]
[Bmim][Cl]	Cellulose	Self-healing e-skin sensitive to force and moisture	[211]
	Silk fibroin	Support normal cell growth and differentiation	[129]
[Bmim][PF_6_]	Poly- N-succinimidyl acrylate (p-NSA)	Glucose sensor	[212]
	Propylene Carbonate	Ammonia biosensor	[203]
[Bmim][BF_4_]	Chitosan	Protein and enzyme sensing platform	[213]
	Propylene Carbonate	Ammonia biosensor	[203]
	2-[[(butylamino)carbonyl]oxy]ethyl acrylate	Touch sensor	[214]
[Bmim][OTf]	Propylene Carbonate	Ammonia biosensor	[203]
[Emim][TFSI]			
[Bmim][PF_6_]	Graphite powder and others	Detection of rosmarinic acid in plant extracts	[215]
[Bmim][BF_4_]			
[VEIm][DCA]	Silica + ammonium persulfate	Self-healing sensor for Breathing detection/analysis	[216]
[Ch][MA]	Levan-Glycerol	Electrolyte-based organic transistor, ECG measurement	[217]
[6MQc][TFSI]	None	Intracellular pH monitoring	[218]

## Data Availability

Not applicable.

## Nomenclature

**Ionic Liquid**	**Name**
[Amim][Br]	1-allyl-3-methylimidazolium bromide
[Amim][Cl]	1-n-allyl-3-methylimidazolium chloride
[Ch][Hex]	choline hexanoate
[Ch][Cit]	choline citrate
[Ch][DHP]	2-hydroxyethyl-trimethylammonium dihydrogen phosphate
[Ch][Cl]	choline chloride
[Cho][Phe]	2-hydroxyethyl)-trimethylammonium-L-phenylalaninate
[Cho][Glu]	2-hydroxyethyl)-trimethylammonium-L-glutaminate
[Ch][MA]	cholinium malonate
[C_2_OHmim][Cl]	1-(2-Hydroxyethyl)-3-methyl imidazolium chloride
[Chol][HSO_4_]	choline hydrogen sulphate
[Emim][OH]	1-ethyl-3-methylimidazolium hydroxide
[Emim][Ac]	1-ethyl-3-methylimidazolium acetate
[Emim][Cl]	1-ethyl-3 imidazolium chlorate
[Emim][TFSI]/[C_2_mim][NTf_2_]	1-ethyl-3-methylimidazolium bis(trifluoromethylsulfonyl)imide
[Emim][PF_6_]	1-ethyl-3-methylimidazolium hexafluorophosphate
[Emim] [BF_4_]	1-ethyl-3-methylimidazolium tetrafluoroborate
[Bmim][BF_4_]	1-butyl-3-methylimidazolium tetrafluoroborate
[Bmim][Ac]	1-butyl-3-methylimidazolium acetate
[Bmim][OAc]	1-butyl-imidazolium acetate
[Bmim][Cl]	1-butyl-3-methylimidazolium chloridev
[Bmim][HSO_4_]	1-butyl-3-methylimidazolium hydrogen sulphate
[Bmim]_2_[NiCl_4_]	bis(1-butyl-3-methylimidazolium) tetrachloronickelate
[Bmim][TFSI]	1-butyl-3-methylimidazolium bis(trifluoromethanesulfonyl)imide
[MIM][Cl]	1-methylimidazolium chloride
[Hmim][HSO_4_]	1-methylimidazolium hydrogen sulphate
[P_66614_][HBO]	trihexyl(tetradecyl) phosphonium 2-(2′-hydroxyphenyl) benzoxazole
[TEA][A]	triethanolamine acetate
[TEA][SO_4_H]	triethylammonium hydrogen sulfate
[TEA][PO_4_H]	triethylammonium hydrogen phosphate
[VAPim][BF_4_]	1-vinyl-3-(3-aminopropyl)-imidazolium tetrafluoroborate
[VHim][NTf_2_]	1-vinyl-3-hexylimidazolium bis(trifluoromethanesulfonyl)imide
[VEIm][DCA]	1-vinyl-3-ethylimidazolium dicyanamide
